# Genome-wide profiling of DNA methylation provides insights into epigenetic regulation of fungal development in a plant pathogenic fungus, *Magnaporthe oryzae*

**DOI:** 10.1038/srep08567

**Published:** 2015-02-24

**Authors:** Junhyun Jeon, Jaeyoung Choi, Gir-Won Lee, Sook-Young Park, Aram Huh, Ralph A. Dean, Yong-Hwan Lee

**Affiliations:** 1 Department of Agricultural Biotechnology, College of Agriculture and Life Sciences, Seoul National University, Seoul 151-921, Korea; 2 Fungal Bioinformatics Laboratory, Seoul National University, Seoul 151-921, Korea; 3 Department of Bioinformatics and Life Science, Soongsil University, Seoul 156-743, Korea; 4 Center for Fungal Pathogenesis, Seoul National University, Seoul 151-921, Korea; 5 Functional Genomics, North Carolina State University, Raleigh, NC 27607, United States of America; 6 Center for Fungal Genetic Resources, Seoul National University, Seoul 151-921, Korea

## Abstract

DNA methylation is an important epigenetic modification that regulates development of plants and mammals. To investigate the roles of DNA methylation in fungal development, we profiled genome-wide methylation patterns at single-nucleotide resolution during vegetative growth, asexual reproduction, and infection-related morphogenesis in a model plant pathogenic fungus, *Magnaporthe oryzae*. We found that DNA methylation occurs in and around genes as well as transposable elements and undergoes global reprogramming during fungal development. Such reprogramming of DNA methylation suggests that it may have acquired new roles other than controlling the proliferation of TEs. Genetic analysis of DNA methyltransferase deletion mutants also indicated that proper reprogramming in methylomes is required for asexual reproduction in the fungus. Furthermore, RNA-seq analysis showed that DNA methylation is associated with transcriptional silencing of transposable elements and transcript abundance of genes in context-dependent manner, reinforcing the role of DNA methylation as a genome defense mechanism. This comprehensive approach suggests that DNA methylation in fungi can be a dynamic epigenetic entity contributing to fungal development and genome defense. Furthermore, our DNA methylomes provide a foundation for future studies exploring this key epigenetic modification in fungal development and pathogenesis.

DNA methylation is a common epigenetic modification of DNA in eukaryotes that has crucial roles in cellular processes including genome regulation and development^1^,^2^,^3^. Eukaryotic DNA methylation occurs almost exclusively in cytosine bases and is usually associated with transposable elements and repeats sequences of many organisms, maintaining repressive chromatin state^3^,^4^. Recently, studies on flowering plants and mammalian stem cells revealed that DNA methylation takes place not only in promoter regions of genes, transposable elements and repeat sequences but also in transcribed regions of genes^4^,^5^,^6^,^7^. In plants and mammals, defect in DNA methylation has been shown to result in pleiotropic morphological abnormalities and embryonic lethality, respectively, attesting to the importance of this modification during developmental processes^8^,^9^. Implication of DNA methylation in coordinating developmental programs of higher eukaryotes is based on global reprogramming of methylation landscape and/or gene imprinting through separable genetic pathways involving maintenance (DNMT1) and *de novo* DNA methyltransferases (DNMT3) during development^9^,^10^,^11^.

In fungi, a number of studies have been conducted to identify DNA methyltransferases (DNMTase) and methylated regions of the genome^12^,^13^,^14^,^15^,^16^,^17^,^18^. Although DNMT1 family of DNMTases was found, no DNMT3 homologues have been found in any fungal genome so far. Notably, ascomycete fungi possessed Dnmt1-related DNA methyltransferase, DIM-2, which methylates transposable elements and repeat sequences in a way that are dependent on methylation of lysine 9 of histone H3^13^,^19^,^20^. A genome-wide study on a model ascomycete fungus, *Neurospora crassa* suggested that fungal DNA methylation is a stable mark for genome defense since it is not found in bodies of structural genes and silences transposable elements and repetitive DNA sequences that have undergone repeat-induced point mutation^21^,^22^,^23^. Recent works on DNA methylation in fungi have reported that gene body methylation is found in some species such as *Candida albicans* and *Uncinocarpus reesii*^18^. However, these studies mostly examined DNA methylation of single cell type or tissue such as fungal hyphae, and thus dynamic nature of DNA methylation during development of fungi has not been investigated at genomic scale to date, although some studies showed changes in overall 5-methylcytosine (5-mC) content during fungal development using methylation sensitive and insensitive restriction enzymes^24^,^25^.

In this study, we set out to study DNA methylation in a model plant pathogenic fungus, *Magnaporthe oryzae.* *M. oryzae* is a causal agent of rice blast disease, which is the most devastating disease in cultivated rice, the staple food for more than half of the human population^26^. It has been estimated that enough rice to feed 60 million people is destroyed annually by the disease^27^. *M. oryzae* usually grows as mycelium and produces a three-celled asexual spore, called conidium as primary source of inoculum. The conidium that lands on leaf surface germinates in presence of water and develops a specialized infection structure called an appressorium. The fungus gains entry into host plant by mechanically rupturing the leaf cuticle using turgor pressure of up to 8 MPa within appressorium, and grows inside the plant tissues siphoning off nutrients, leading to yield losses or even death of infected plant^28^.

Despite much progress made in unraveling and understanding genetic pathways that govern morphogenesis in *M. oryzae*^29^,^30^,^31^, the contribution of epigenetic factors such as DNA methylation to this fundamental process has yet to be thoroughly investigated. In *M. oryzae*, existence of DNA methylation was examined by restriction enzyme-based work on a retrotransposon, MAGGY^32^. Genome-wide distribution and implication of DNA methylation in life cycle of the fungus is not known. Here we combined whole-genome bisulphite-sequencing (BS-seq) and genetic manipulations in order to understand the mechanism of DNA methylation and to profile genome-wide patterns of DNA methylation during fungal development at single nucleotide resolution. Our work uncovered global changes and genetic basis of DNA methylation across the genome of *M. oryzae* during development. To expand on these observations, we examined the impact of DNA methylation on transcriptional activity of fungal genome through RNA-seq. Our unbiased, genome-wide approach provides insight into the dynamic nature of DNA methylation and its implication in development of a plant pathogenic fungus.

## Results

### Whole-genome bisulphite sequencing of the fungal methylomes

As a first step to probe into DNA methylation during development of *M. oryzae*, we generated genome-wide DNA methylation maps across the fungal genome by conducting high throughput bisulphite sequencing (BS-seq) for genomic DNAs extracted from mycelia, conidia, and appressoria (Supplementary Table S1). BS-seq of these samples yielded about 19–21 million reads corresponding to sequence output ranging from 1.73 to 1.89 Gbp per sample after filtering. Lambda DNA, which lacks DNA methylation, was included in bisulphite treatment of samples to estimate conversion rate. In our study, conversion rate was at least 99.33%. To minimize the bias that may result from unequal number of reads per sample, ~18.9 million reads (1.7 Gbp) were chosen at random from each sample. Among these reads, 72.65 to 81.57% (except for *ΔMorid* sample showing 62.75% mapping rate) of reads were successfully aligned to the converted reference genome, resulting in 30.1 to 33.8 × coverage of the 41 Mbp genome, the highest reported in fungi to date. In our data, over 97% of total cytosines present in *M. oryzae* genome was covered by at least, one sequencing read and more than 88% of cytosines were covered by at least four sequencing reads, allowing methylation level of individual sites to be determined with reasonable confidence. BS-seq data were validated by cloning and sequencing PCR products from bisulphite-treated DNA (BS-PCR)^33^,^34^ for six loci predicted to carry methylation or no methylation (Supplementary Table S2). Comparison of two independent datasets for six loci showed that over 90% of mCs identified in our sequencing results overlapped with mCs in BS-seq data, pointing out sensitivity and reliability of our data (Supplementary Table S2 and Supplementary Figure S1).

### Genome-wide DNA methylation pattern in the genome of *M. oryzae*

**Figure 1:**
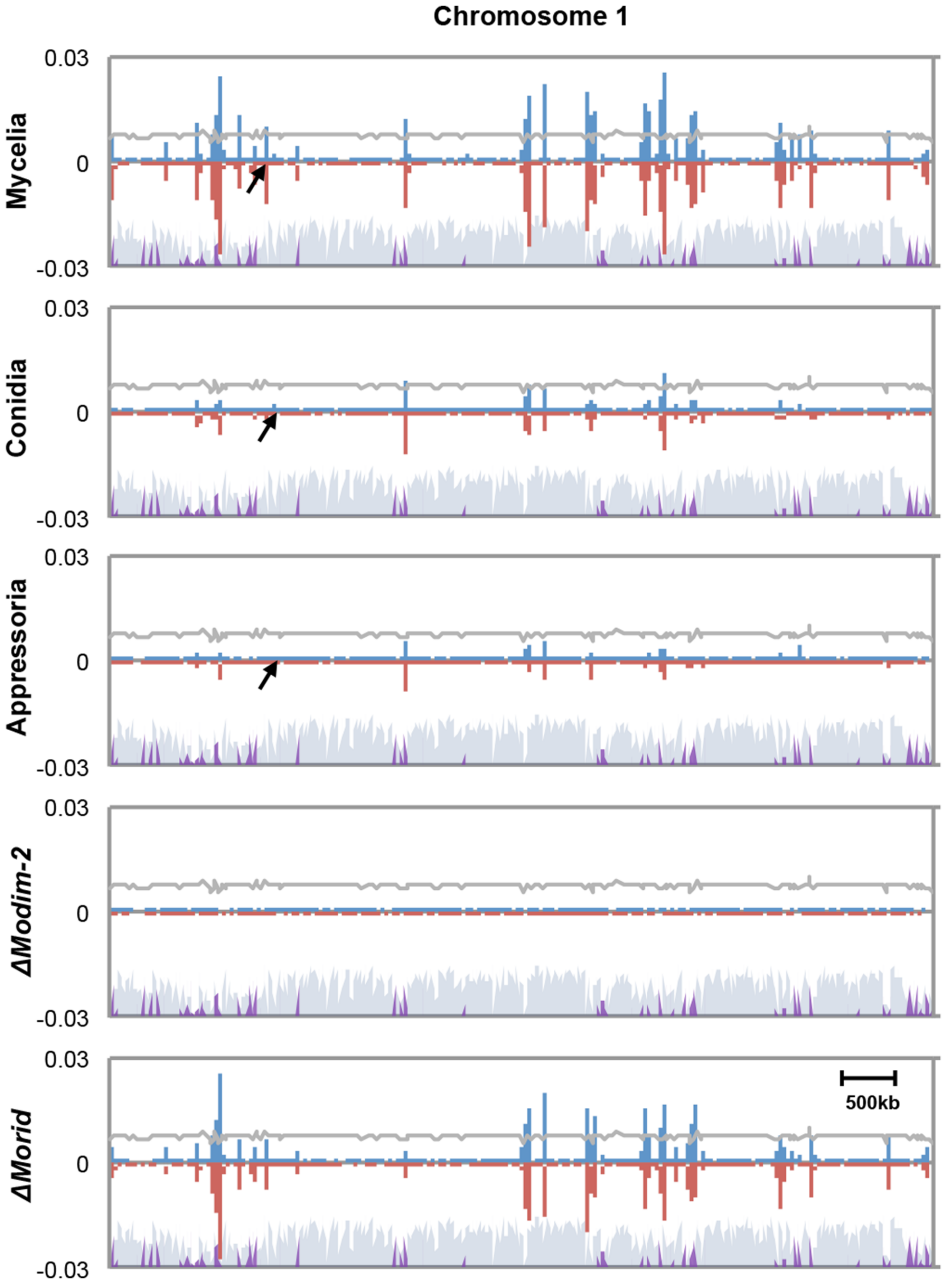
Chromosomal distribution of DNA methylation in the genome of *M. oryzae*. The density of methylcytosines (mCs) identified on each strand throughout chromosome 1 (supercontig 8.1) of each sample was calculated and plotted in 10 kb bin. Blue and red bars indicate methylation densities in Watson and Crick strands, respectively. Gray and purple shades on the bottom indicate density of genes and transposable elements, respectively. Arrows indicate the region that is magnified in Figure 4C.

From our BS-seq analysis, varying number of methylated cytosine (mC) sites was identified in the genome of each sample, while the average methylation level (defined as number of mC reads divided by number of total reads covering the site) of individual mC sites remained at between 20 and 30% across chromosomes and samples (Supplementary Figure S2A and S2B, and Supplementary Dataset 1–4). A total of 46,124 mC sites were identified in mycelia, accounting for ~0.22% of all genomic cytosines. In mycelia, mC sites were found in all sequence contexts with strong preference to CG and CHH contexts (H: A, T, and C) (Supplementary Figure S2A). Notably, mC sites in mycelia were not evenly distributed but clustered across chromosomes, forming densely methylated domains around transposable element (TE)-rich and gene-poor regions (Figure 1 and Supplementary Figure S3). DNA methylation density showed high correlation (Pearson correlation coefficient = 0.79) with density of transposable elements across the chromosomes, supporting that TEs are one of major substrates of DNA methylation in the fungus (Supplementary Figure S4). Those methylated domains shrunk dramatically in conidia (14,530 mC) and appressoria (11,585 mCs) (Figure 1, Supplementary Figure S3 and Supplementary Dataset 1–4). This result suggests that DNA methylation of *M. oryzae* undergoes changes with progression of development.

### Identification of genes encoding putative DNA methyltransferases

**Figure 2:**
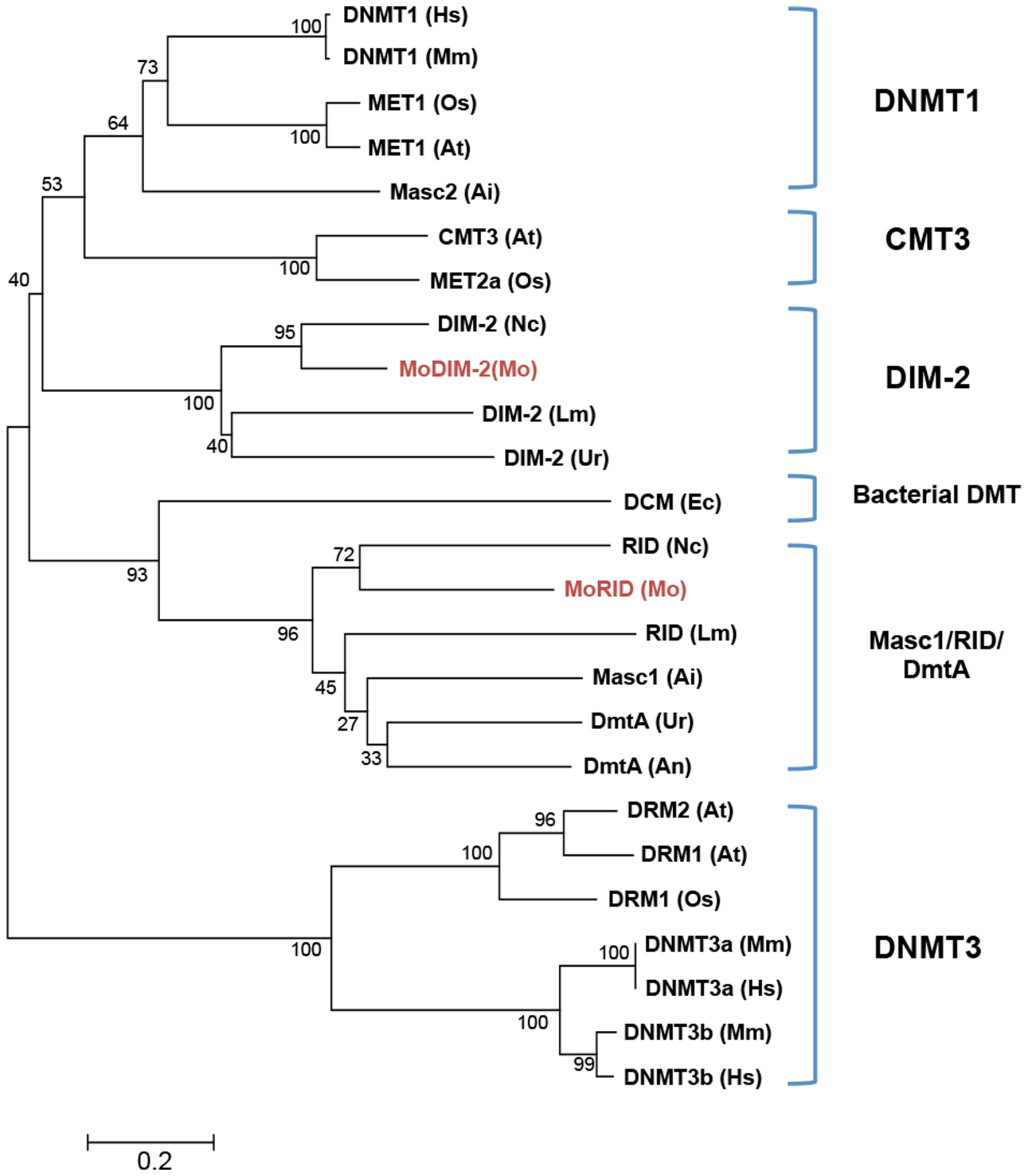
Phylogenetic analysis of DNA methyltransferases. Phylogenetic tree was constructed by neighbor-joining method using DNA methyltransferase domain of representative amino acids sequences of DNA methyltransferases. Hs, *Homo sapiens*; Mm, *Mus musculus*; At, *Arabidopsis thaliana*; Os, *Oryza sativa*; Ai, *Ascobolus immerses*; Lm, *Leptospheria maculans*; Ur, *Uncinocarpus reesii*; Nc, *Neurospora crassa*; Mo, *Magnaporthe oryzae*; Ec, *Escherichia coli*.

To further investigate the observed correlation between DNA methylation and development in *M. oryzae*, we sought to identify and characterize genes encoding putative DNMTases in the fungal genome. Through BLAST search using amino acid sequences of known DNMTases as queries, we found two genes encoding proteins containing the DNMTase domain (MGG_00889 and MGG_02795). Among DNMTase families found in eukaryotes, DNMT1 and DNMT3 families are involved in maintenance and *de novo* DNA methylation, respectively, while DNMT2 was shown to be a tRNA methyltransferase and DNMT4 includes Masc1, a novel *de novo* DNMTase and RID, a protein involved in repeat induced point mutation (RIP)^12^,^13^,^15^,^35^,^36^,^37^,^38^. Our phylogenetic analysis showed that two DNMTase domain-containing proteins (MoDIM-2 and MoRID) are closely related to DIM-2 and RID of *N. crassa*, respectively (Figure 2). Such presence of the two types of DNMTases is common in most of fungal species in Pezizomycotina (Supplementary Figure S5A). These data indicate that *M. oryzae* has a potential DNMTase homologous to DIM-2 but no specialized *de-novo* DNMTase. In accordance with this, domain architecture analysis of DNMTases showed that MoDIM-2 bears long N-terminal extension containing BAH (bromo-adjacent homology) domain and C-terminal extension, typical of DIM-2 type of fungal DMTases, but does not have PWWP (proline-tryptophan-tryptophan-proline) domain commonly found in DNMT3 family (Supplementary Figure S5B).

### Transcriptional profiling of two putative DMTase-encoding genes

**Figure 3:**
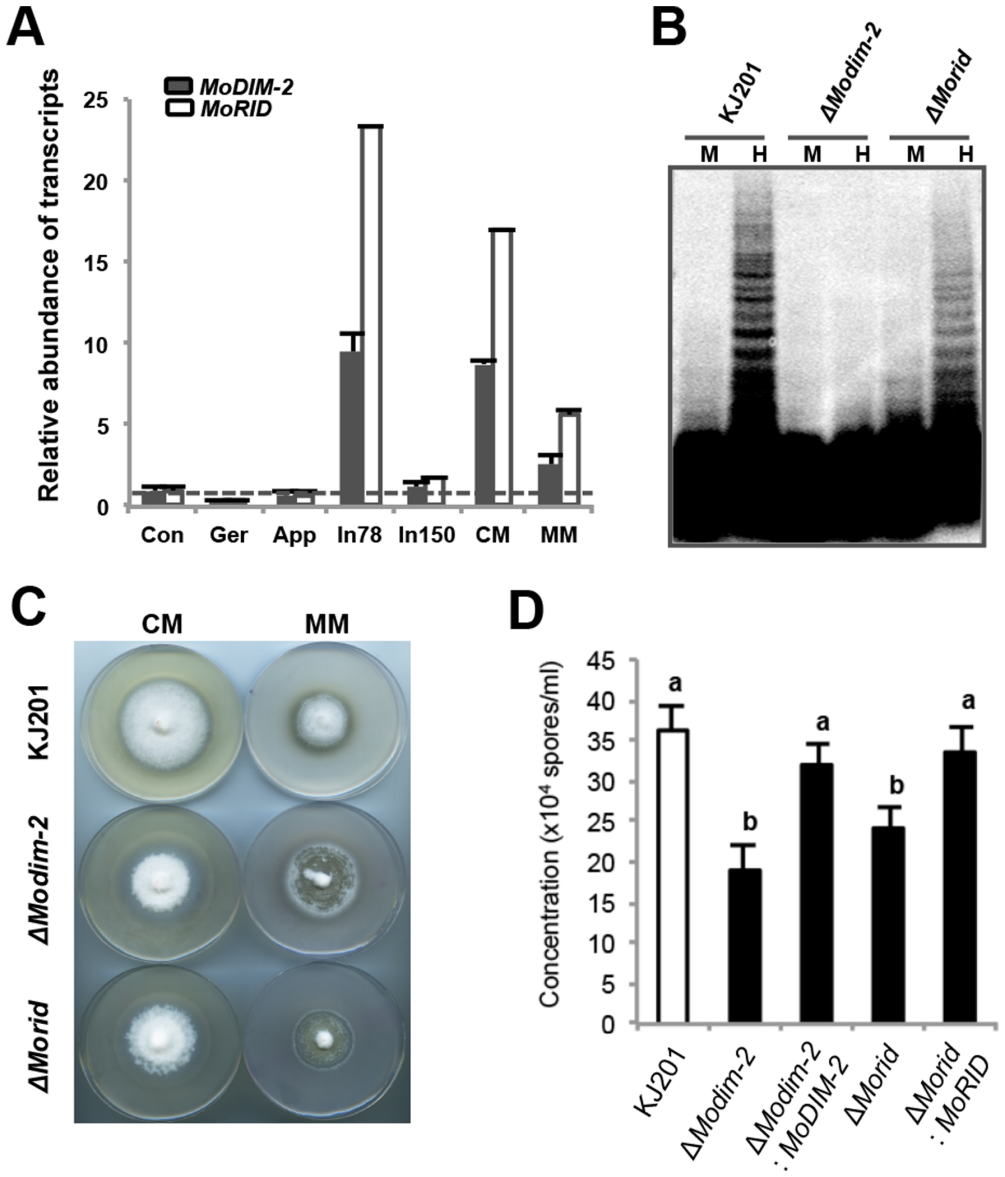
Genetic analyses of DNA methyltransferases in a model plant pathogenic fungus, *Magnaporthe oryzae*. (A) Expression profile of DNA methyltransferase genes using real-time PCR. Relative abundance of transcripts was normalized to conidia. Con, conidia; Ger, germination; App, appressoria; In78, infected leaves 78 hours post inoculation (hpi); In150, infected leaves 150 hpi; CM, complete media; MM, minimal media. (B) Southern blot analysis of MAGGY methylation in wild-type and mutants using isoschizomer. M, *Msp*I; H, *Hpa*II. The image is showing entire blot with only upper (including a part containing loading wells) and bottom margins removed for clarity. (C) Vegetative growth on complete and minimal media (CM and MM, respectively). (D) Asexual reproduction in the mutants. Results are mean ± SD (Tukey test, P < 0.05).

When relative abundance of *MoDIM-2* and *MoRID* transcript during vegetative growth and fungal development was analyzed, transcription of *MoDIM-2* and *MoRID* showed high degree of correlation across all the conditions used for analysis (Pearson correlation coefficient = 0.988), suggesting the same or overlapping transcriptional network for regulation of two genes (Figure 3A). Compared to hyphal growth conditions (CM, MM and In78), expression of the two genes were down-regulated in conidia, germinating conidia, appressoria and 150 hours post inoculation (In150), suggesting that hyphal growth is positively associated with transcriptional regulation of two genes. This expression pattern is consistent with the observed reduction of mC sites during development, suggesting the possibility of DNA methylation being regulated at transcriptional level (Figure 1 and Figure 3A).

### Genetic analysis of DNA methyltransferases and methylomes in *M. oryzae*

To genetically test whether the two putative DNMTase genes are involved in DNA methylation, we generated deletion mutants of individual genes (Supplementary Figure S6A) and checked the DNA methylation status of a known methylated retrotransposon, MAGGY in mycelia of the mutants via Southern hybridization using methylation-sensitive and -insensitive restriction enzyme isoschizomers (Figure 3B and Supplementary Figure S6B). This analysis showed that there is no or little methylation in MAGGY within the genome of Δ*Modim-2* and considerable but lower methylation than wild-type strain KJ201 within the genome of Δ*Morid*. This result indicates that *MoDIM-2* is a major player in DNA methylation of MAGGY and that *MoRID* has some but limited roles in DNA methylation. We further confirmed the role of two putative DNMTases by whole genome bisulphite sequencing of the mutant genomes (Figure 1). In contrast to the wild-type with 46,124 mC sites, in *ΔModim-2*, only 4,563 mC sites were found, while in *ΔMorid*, 36,809 cytosine sites remained methylated, providing genomic scale evidence for MoDIM-2 as a major DNA methyltransferase in the fungus. In addition, ~25% of remaining mC sites in *ΔMorid* didn't overlap with those in wild-type, suggesting the possibility of *MoRID* in regulating methylation specificity. In *N. crassa*, *RID*, an ortholog of *MoRID*, is considered to have no DNMTase activity. Therefore, we investigated the observed reduction of mC sites in *ΔMorid*, compared to the wild-type. We found that all the mC sites that disappeared in *ΔMorid* were a subset of missing sites in *ΔModim-2*. This observation suggests that methylation of those cytosine sites requires both *MoDIM-2* and *MoRID*.

### Impact of disrupting DNA methylation on fungal life cycle

Next we asked if disruption of DNA methylation has any impact on life cycle of *M. oryzae* by examining phenotypic changes of mutants. First, we observed abnormalities in colony morphology of Δ*Modim-2* (Figure 3C). The radial growth of the mutants was indistinguishable from that of wild-type, but the mutant colony was fluffier than wild-type. Difference in colony morphology was most dramatic in minimal media where the mutant showed premature autolysis phenotype. Notably, a significant reduction in production of conidia for both mutants and a delay in appressorium formation for *ΔModim-2* were also observed with no apparent defect in morphology of conidia and appressoria (Figure 3D, Supplementary Figure S6C and S6D). However, all the mutants retained the ability to cause disease on rice plants (Supplementary Figure S6E). Taken together, our analysis of DNMTase genes in *M. oryzae* suggests that *MoDIM-2*- and/or *MoRID*-mediated DNA methylation is important in normal development of the fungus but is dispensable for pathogenicity.

### DNA methylation in genomic features during development in *M. oryzae*

**Figure 4:**
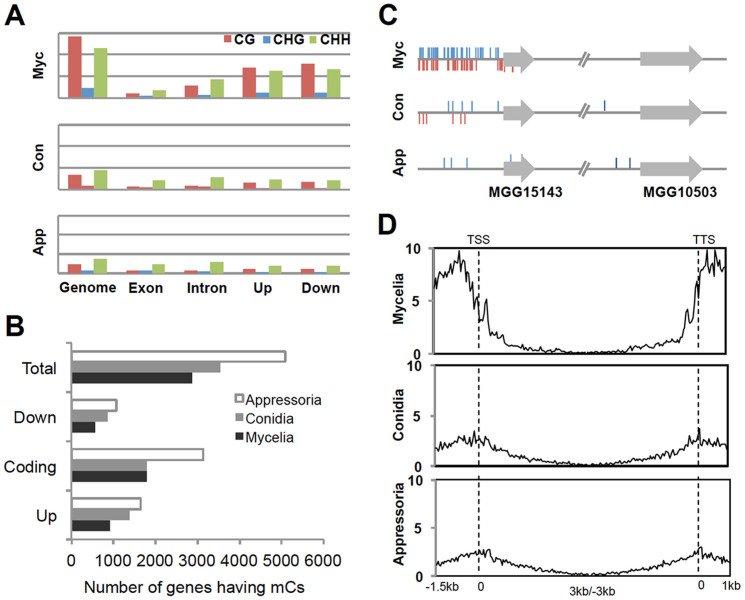
Dynamics of DNA methylation in the genome of *M. oryzae*. (A) Density of mCs in different genomic features, counted in total length of corresponding feature (multiplied by 10^4^). Up and Down indicate 1.5 kb upstream and 1 kb downstream regions of annotated genes, respectively. (B) Number of genes having DNA methylation in upstream, downstream, and coding sequences during development. To avoid over-counting issue, assignment of mC sites to genomic categories was done based on proximity to genes near it. For example, if a mC site is at downstream of locus A and at the same time upstream of locus B but closer to locus B, then this site was counted as upstream methylation of locus B. (C) A representative region of chromosome 1 (indicated by arrows in Fig. 1) showing dynamic changes in DNA methylation during development. (D) Density of mCs in and around genes associated with DNA methyaltion during fungal development. Genes were aligned at the 5′ end (left dashed line) or the 3′ end (right dashed line) and number of mCs in each 50 bp interval was plotted across genic region.

Given the importance of DNA methylation, we were determined to further explore the distribution of mCs in different genomic features, by calculating and comparing DNA methylation densities of whole genome, genes (exons and introns), and upstream (1.5 kb) and downstream (1 kb) of genes (Figure 4A and Supplementary Dataset 1–4). Compared to the whole genome, the density of mCs in genic regions (defined as upstream + exon + intron + downstream) was low. This is probably due to DNA methylation of transposable elements and repeated sequences within intergenic regions. Nevertheless, a significant proportion of mC sites were found in genic regions (2,868 genes in mycelia) (Supplementary Table S3). We designated such genes having mCs in genic regions as mORFs. Since overall number of mC sites decreases during development, we expected that the number of mORFs would concomitantly decrease. Contrary to our expectation, however, we found that the number of mORFs increased in conidia and appressoria (Figure 4B). In-depth analysis of this apparent contradiction revealed that fungal methylome undergoes global reprogramming during development: the overall reduction in mC sites during development was not simply the result of loss of methylation in pre-existing mC sites but of dynamic process by which losses of previous mC sites were accompanied by gains of new mC sites in different loci (Figure 4C).

Within genic regions, we found that methylation peaks in the upstream and downstream regions of genes, showing a sharp drop at the boundaries of coding sequences (Figure 4D). The methylation peaks in the regions flanking coding sequences in mycelia disappeared in conidia and appressoria. Gene body methylation occurred mostly near the start and the end of coding sequences and was depleted at the center. This change in DNA methylation profile in and around genes suggests that DNA methylation may play regulatory roles in transcription of mORFs.

### Effect of DNA methylation on transcript abundance of genes

**Figure 5:**
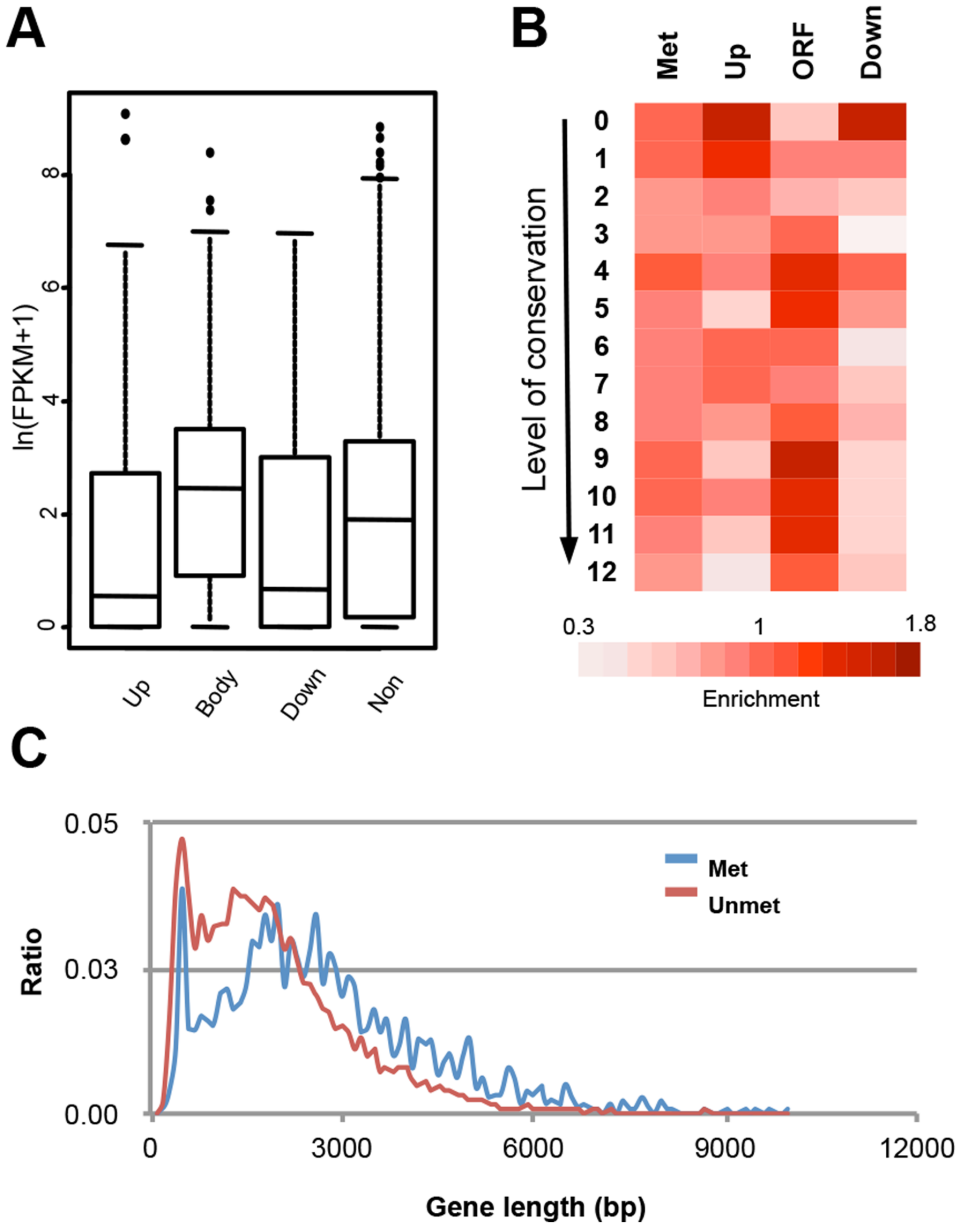
Relation between DNA methylation and transcription. (A) Transcript abundance of mORFs and position of mCs. Up, upstream regions; Body, gene body; Down, downstream regions; Non, non-methylated genes. (B) Conservation level of mORFs and position of mCs. Conservation of a gene was measured by counting number of species where ortholgue(s) is found for a given gene throughout 13 species in Pezizomycotina (Figure S5). Proportion of genes that fall in each conservation category was shown relative to non-methylated genes. (C) Gene length distribution for genes with and without body methylation (blue and red lines, respectively).

We next evaluated the global impact of DNA methylation on transcriptional activity of the genome via RNA-seq. To separate the effect of DNA methylation on transcriptional consequences from the effect of developmental cues, we performed RNA-seq for total RNAs extracted from mycelia of wild-type, Δ*Modim-2*, and Δ*Morid*. Levels of steady-state transcript for both mutants were generally similar to that of wild-type, although we identified about 1.2% and 2.4% of annotated genes with altered transcript abundance (≥2-fold difference relative to wild-type) in *ΔModim-2* and *ΔMorid*, respectively. Despite not so dramatic changes in transcription between wild-type and mutants, we detected differences in transcript abundances depending on the context of genes in which methylation occurs (Figure 5A). mORFs with methylation in upstream (^up^mORF) or downstream regions (^down^mORF) tend to have lower transcript abundance than genes with no methylation. The genes with no methylation tend to have lower transcript abundance than genes having body methylation (^body^mORF) (Kolmogorov–Smirnov test, *P* < 1 × 10^−8^).

The observed dependency of transcription on DNA methylation led us to check if mORFs show enrichment in specific functions. The vast majority of mORFs were, however, either predicted or hypothetical proteins to which biochemical functions could not be assigned (Supplementary Dataset 1–4). As an attempt to infer the biological significance of mORFs, we classified the mORFs into twelve groups based on their conservation throughout 13 fungal species including *M. oryzae* (Supplementary Figure S5A) in Pezizomycotina using InParanoid^39^. Intriguingly, ^up^mORFs and ^down^mORFs showed low conservation level, while ^body^mORFs tended to be highly conserved (Figure 5B). Recently proposed *de novo* gene birth mechanism suggests that high conservation of genes may be indicative of their origin or birth dating farther back in evolution, compared to the ones exhibiting no or weak conservation^40^. One of the predictions of the proposal is that gene of high conservation should be longer than gene of weak conservation. Consistent with this prediction, mean length of ^body^mORFs was ~2.37 kb, which is longer by 23% than the mean length of genes with no body methylation (~1.92 kb) in mycelia (*P* < 2.2 × 10^−16^, based on permutation test) (Figure 5C).

### Transposable elements are differentially methylated and additional layer of regulation may exist for suppressing their transcription

**Figure 6:**
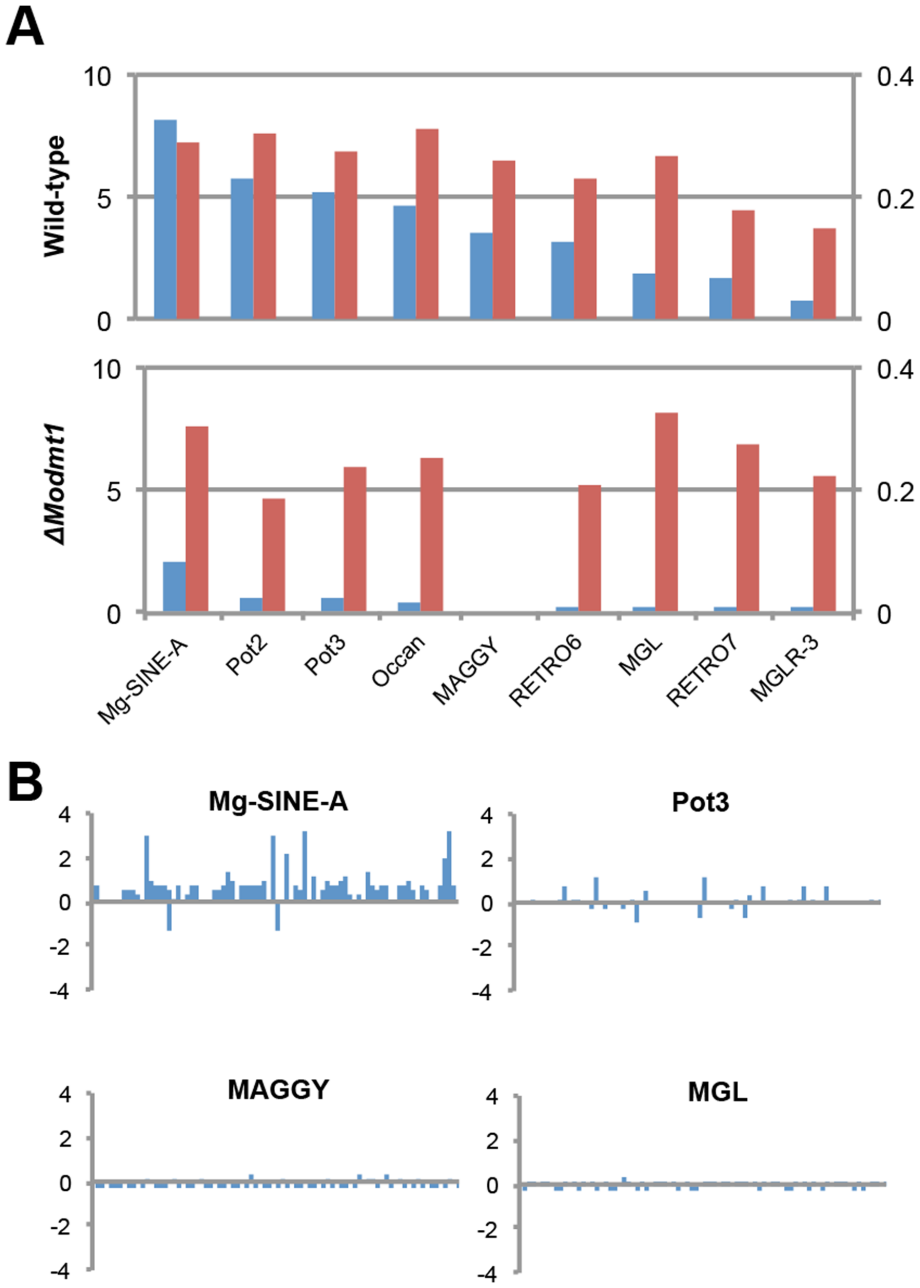
DNA methylation and transposable elements (TE) in *M. oryzae*. (A) Density of methylcytosines (number of mCs/length of each TE in kb) (blue bar and Y-axis on the left) and average methylation level (red bar and Y-axis on the right) of TEs in wild-type and *MoDIM-2* deletion mutant. The names of TEs are arranged on X-axis according to their length in ascending order. (B) RNA-seq analysis of TE expression in *ΔModim-2*, relative to wild-type strain KJ201 (log_2_(*ΔModim-2/*KJ201)). Individual bars in x-axis represent TEs in different genomic locations.

We analyzed DNA methylation of transposable elements (TEs) in *M. oryzae* apart from our analysis on mORFs. To date, relationship between fungal DNA methylation and transposable elements have not been scrutinized at high resolution. Using our bisuphite-sequencing data, first, we inquired into methylation density and level of TEs at genomic scale. For nine types of TEs whose defining sequences are available and not too short (>400 bp), the density and average methylation level of mCs sites in each type of TE were examined. Our analysis uncovered that TEs are differentially methylated by MoDIM-2 in a manner that negatively correlates with the length of TEs (Figure 6A and Supplementary Figure S7). Remaining DNA methylation in Δ*Modim-2* suggests involvement of *MoRID* in methylation of those sites.

DNA methylation is considered to target TEs for transcriptional silencing. If DNA methylation were sufficient to suppress transcription of TEs, transcription of TEs would increase in Δ*Modim-2*. When transcription of TEs was compared between wild-type and Δ*Modim-2*, changes in transcript abundance of TEs displayed diverse patterns, depending not on presence of DNA methylation but on type and genomic location (Figure 6B). In cases like Mg-SINE-A, however, most of TEs were up-regulated in Δ*Modim-2*. This suggests that DNA methylation is a mechanism of suppressing transcriptional activity of TEs as in other organisms but there are additional layer of regulation on transposable elements.

## Discussion

Over the past decade, genome-wide analysis of DNA methylation was carried out in a number of fungal species^17^,^18^,^21^. However, those studies have primarily focused on identification and analysis of DNA methylation sites across fungal genomes from a single experimental condition. Here, we reported profiling of fungal methylomes from multiple conditions: mycelia, conidia, and appressoria of the rice blast fungus, *M. oryzae*. The results of this study, to our knowledge, represent the first DNA methylomes of plant pathogenic fungus and provide an overview of methylation dynamics during fungal development. Our comparative analysis of methylomes showed that mycelia are the tissue with the highest number of methylated cytosine and that significant proportion of these methylation sites disappears in conidia and appressoria. In plants and mammals, it has been demonstrated that there exist multiple active demethylation pathways, some of which involve DNA glycosylase^41^. Although *M. oryzae* has a gene encoding putative DNA glycosylase in its genome, it is not clear whether this putative DNA glycosylase has any roles in DNA demethylation. Our analysis of transcription pattern of DNMTase genes suggests that the observed reduction of DNA methylation is likely a product of passive demethylation of mycelial methylome by down-regulating these DNMTase genes in the course of conidiogenesis and appressorium formation.

In mycelia, we observed that ~0.22% of total cytosine sites in the genome were methylated and average methylation level at mC sites is ~30%, regardless of tissue types examined. These methylation statistics are within the range of values observed in other fungi that show varying low levels of DNA methylation. In *Aspergillus flavus*, only 0.04% of total cytosine sites and less than 5% methylation level were observed in two biological replicates of BS-seq. Furthermore, BS-PCR failed to reproduce the methylcytosines from BS-seq experiments, leading to the conclusion that DNA methylation is negligible and may transiently occur during obscure sexual stage of this fungus^17^. However, when BS-PCR to validate the methylation status was carried out in our work, most of methylation sites were shared between the whole-genome bisulphite sequencing and BS-PCR datasets, implying that methylation sites observed in this study are bona fide ones and unlikely a false positive resulting from incomplete conversion reaction.

A consensus view on DNA methylation in fungi is as a genome defense mechanism against transposable elements and repeats sequences. In consistent with this view, TEs within genome of *M. oryzae* were highly methylated. However, our work yielded two new observations on relationship between TEs and DNA methylation. First, TEs are differentially methylated in density, depending on their length. In *N. crassa*, RIP is known to take place during sexual reproduction in repeated sequences that are greater than ~400 bp^42^,^43^. Those sequences that underwent RIP are mostly associated with methylated DNA^44^. Since the shortest TE we used in our analysis, Mg-SINE-A is 472 bp long and sexual stage of *M. oryzae* has not been reported in the field, RIP is not likely to be a factor influencing length dependency of TE methylation. Currently more data should be forthcoming to explain such negative correlation between TE length and DNA methylation density. Second, many of TEs are not transcriptionally activated upon demethylation in *MoDIM-2* deletion mutant. In general, DNA methylation is preceded by histone methylation, suggesting that DNA methylation leads to stable long-term heterochromatic state, compared to readily reversible histone methylation^45^. It may be possible that histone methylation is an additional layer of regulation for transposable elements. The fact that TEs in yeast lacking DNA methylation is under tight control supports the possibility of histone methylation playing a part in regulation of TEs in *M. oryzae*.

In addition to methylation in TEs, about 20% of non-TE genes are also methylated in mycelial nuclei of the fungus. Together with recent studies showing gene body methylation in fungal species including *C. albicans*, our observation suggests that gene body methylation may be more common than previously thought in the fungal kingdom. Interestingly, we found that DNA methylation patterns undergo genome-wide changes in a way that correlate with progression of pathogenic development in *M. oryzae*. Such dynamic nature of DNA methylation was not reported in filamentous fungi before. Our data suggested that DNA methylation dynamics are regulated by DNMTases at transcriptional level. Disruption of this regulation by deleting either of DNMTase genes resulted in the fungus defective in normal development, indicating importance of maintaining proper DNA methylation patterns in life cycle of *M. oryzae*. In particular, significant reduction of asexual reproduction in DNMTase gene deletion mutant suggests possible ecological impact of DNA methylation, considering multi-cyclic nature of the disease. However, in this study, genes that have been known to play roles during conidiation and/or appressorium formation were found to have no or negligible DNA methylation in all tissues examined. Such near absence of DNA methylation in genes of major effects may explain the lack of dramatic phenotypes in deletion mutants of DNMTase genes from current and previous study aimed at analysis of DNA methylation in *M. oryzae*^46^. Only less than 1% of genes in the genome were shown so far to have major impact on fungal development. Moreover, it is very likely that genes having subtle effects on morphogenetic processes have been ignored due to lack of sensitive methods. We argue that the absence of known genes in our data is not the evidence of absence of roles that DNA methylation may play during fungal development.

In *N. crassa*, both symmetric and asymmetric methylations are propagated by one of DNA methyltransferase domain-containing proteins, DIM-2^13^. The other protein, RID is considered to have no DNA methyltransferase activity. Combining genetic analysis with genome-wide bisulphite sequencing, we revealed that an ortholog of DIM-2, MoDIM-2 is responsible for ~91% of all cytosine methylation and an ortholog of RID, MoRID is involved in methylation of some of the cytosine sites in *M. oryzae*. Importantly, deletion of *MoRID* gene changed the position of significant portion (~25%) of methylated cytosines that were present in wild-type, implying interaction or cooperation between MoDIM-2 and MoRID for substrate specificity. It has not been demonstrated that MoRID is required for repeat induced point mutation (RIP) in *M. oryzae* as its ortholog is in *N. crassa*, although there is evidence for ongoing RIP in the genome of *M. oryzae*^47^.

**Figure 7:**
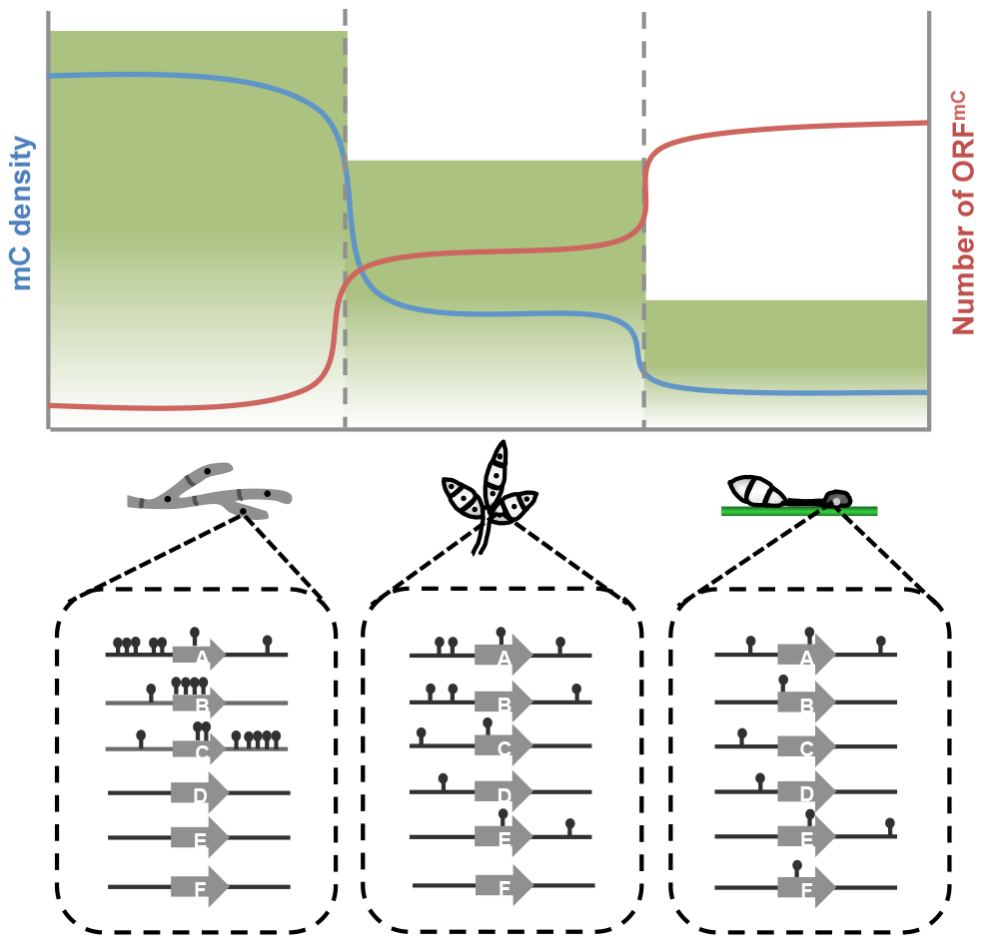
Model for dynamic changes in DNA methylation during fungal development. Height of green shade represents degree of developmental potential of fungal cells. Blue and red lines represent density of mCs and number of genes associated with mCs (ORF^mC^) during development. Schematic diagram below the graph describes dynamic reprogramming of DNA methylation in and around genes within nuclear genomes of mycelia, conidia, and appressoria.

It is worth noting that DNA methylation dynamics in *M. oryzae* has intriguing parallel to mammalian DNA methylation in that developmental potency of cells is connected to DNA methylation. In mammals, it has been established that DNA methylation is reprogrammed in a way that genome-wide demethylation is followed by lineage-dependent *de-novo* methylation as stem cells differentiate into lineage-committed cells^4^,^48^,^49^. This reprogramming allows cells to maintain their identity by ensuring differential transcriptional programs in each cell type. Such regulatory roles of DNA methylation in gene expression of higher eukaryotes during development is thought to be exapted during eukaryotic evolution from its ancestral role in genome defense against invasive elements such as transposons^50^. In *M. oryzae*, mycelia can develop into any cell type, whereas conidia can either grow as vegetative mycelia or develop appressoria after germination, and appressoria has only one cellular fate-host penetration. Our results show that as the developmental potency of a fungal cell becomes restricted, the genome is subjected to massive loss of existing mC sites and simultaneous *de-novo* methylation of unmethylated sites, leading to an opposing pattern between total mC content and number of mORFs (Fig. 7). This cell type-dependent pattern of DNA methylation in *M. oryzae* suggests that fungal DNA methylation, like its mammalian counterpart, may have acquired new roles other than controlling the proliferation of TEs.

DNA methylation was generally regarded as a silencing epigenetic mark. Recent genome-scale mapping of DNA methylation in mammals and plants suggests that the relationship between DNA methylation and transcription is more nuanced than previously realized^51^. Our data showed that density of DNA methylation associated with genes dramatically changes during development. To examine the impact of such changes in transcriptional activity of mORFs, we employed RNA-seq for mycelia of wild-type and deletion mutants of DNMT genes. Analysis of RNA-seq data showed that transcript abundance of mORFs is related to the position of DNA methylation in and around genes: methylation in upstream and downstream regions of ORF has negative effects on transcript abundance, while gene body methylation has positive effects. It is of note that the position of DNA methylation might be associated with evolutionary history of genes (Fig. 5B). However, deletion of DNMT genes, which reduces or abolish methylation, didn't drastically change the transcriptional output of the genome. This may be because transcriptional network that is dependent on DNA methylation is a fine-tuner rather than on-off switch of transcriptional activity. Alternatively, it is possible that changes in DNA methylation pattern requires development-specific factor(s) to be translated into changes in transcription, leading to development-dependent modulation of transcription. Impairment of normal development in DNMT gene deletion mutants supports potential role of DNA methylation in choreographing the complex changes in gene expression that occurs during development.

Our study presents the genome-wide DNA methylation map of a fungal plant pathogen at single nucleotide resolution. We showed that DNA methylation within *M. oryzae* genome occurs in non-TE genes as well as transposable elements. More importantly, we uncovered that DNA methylation pattern changes during pathogenic development and that proper regulation of DNA methylation is important in normal development. These findings have broad implications because contribution of DNA methylation to diverse morphogenetic processes of fungal species has been nearly unappreciated to date. Considering the lack of model system to study DNA methylation in plant pathogenic fungi, our study provides a compelling model in which epigenetic modifications of DNA can be investigated in conjunction with development of microbial eukaryotes. Taken together, our work provides not only new insights into evolution and function of DNA methylation in eukaryotes but also basis to expand our perspective on regulation of fungal development.

## Methods

### Strains and culture conditions

*M. oryzae* strain KJ201 was used as wild-type throughout this work. Wild-type strain and all the mutants generated in this study were maintained on either V8 (4% V8 juice) or oatmeal (50 g of oatmeal per liter) agar plates at 25°C under continuous fluorescent light to promote conidiation. Selection of hygromycin- or geneticin-resistant mutants were performed using TB3 media supplemented with 200 μl ml^−1^ hygromycin B or 800 μl ml^−1^ geneticin. For genomic DNA and total RNA extraction purpose from mycelia, fungus was cultured in complete media (0.6% yeast extract, 0.6% casamino acid, 1% glucose) for three days. Appressorium induction for genomic DNA extraction was done in bulk on hydrophobic side of green-mirror using conidia harvested from oatmeal agar plate culture. For enrichment of appressorial nuclei during sampling process, green-mirror plates on which appressorium formation rate is less than 95% were excluded in sampling.

### DNA isolation and Southern hybridization

Genomic DNA was isolated using two methods depending on follow-up experiments. Genomic DNA for Southern hybridization and bisulphite sequencing purpose was prepared by standard method^52^. Genomic DNA from mutants for PCR–based screening was isolated by ‘quick and safe method'^53^ as follows: a patchy of mycelial mat on TB3 media was taken using sterilized tooth-picks and ground in 1.5 ml e-tube with 500 μl of extraction buffer (100 mM Tris-HCl, 10 mM EDTA and 1 M KCl). Cell debris was removed by centrifugation at 5,000 rpm for 15 min and genomic DNA was pelleted by isopropyl alcohol. The pellet was washed with 70% alcohol and dissolved in strerilzed-distilled water.

Restriction enzyme digestion, agrose gel separation, cloning and DNA gel blotting were performed following the standard protocols^52^. Hybridization was carried out in solution containing 6 × SSC, 5 × Denhardt's solution, 0.5% SDS and 100 μl ml^−1^ denatured salmon sperm DNA, at 65°C. Blots were exposed to phosphoimager analyzer (BAS-2040, Fuji Photo Film Co., Ltd., Tokyo, Japan) and visualized by phosphoimager analyzer software.

### Phylogenetic analysis of DNA methyltransferases

Phylogenetic relationships among DNA methyltransferases were inferred using neighbor-Joining method^54^. The bootstrap consensus tree inferred from 250 replicates is taken to represent the evolutionary history of the taxa analyzed. The evolutionary distances were computed using the JTT matrix-based method and are in the units of the number of amino acid substitutions per site. All positions containing gaps and missing data were eliminated from the dataset (complete deletion option). Phylogenetic analyses were conducted in MEGA4^55^.

### Targeted disruption of genes encoding DNA methyltransferases and their complementation

Targeted disruption of a gene was achieved by transformation of fungal protoplasts with knockout construct containing flanking sequences (~1 kb in length for both 5′ and 3′ flanking) of a target gene and selectable marker. Knockout construct was prepared using modified double-joint PCR^56^. PCR products were directly used for transformation of fungal protoplasts. The mutants were further confirmed by Southern blot analysis. Complementation experiment of knockout mutants were carried out by amplifying target gene and flanking sequences (~1 kb on each side) from wild-type and introducing the resulting PCR products along with pII99 vector harboring geneticin resistant marker into the mutant protoplasts. Geneticin resistant colonies were selected and subsequently screened by PCR for the presence of target gene. Primers used in preparation of knockout construct were listed in Table S3.

### Real-time PCR analysis of DNA methyltransferase expression

Total RNAs were extracted from powder into which cells and tissues from different developmental are ground, using Easy-spin™ total RNA extraction kit (iNtRON Biotechnology, Seoul, Korea). For each sample, 2 μg of total RNA was reverse transcribed into first strand cDNA synthesis with ImProm-II™ Reverse Transcription System (Promega, Madison, WI). Real-time PCR were performed in a 20 μl volume containing 2 μl of cDNA (25 ng of input RNA), 100 nM of each primer and 12.5 μl of 2 × Power SYBR® Green PCR Master Mix (Applied Biosystems, Warrington, UK). Reaction was run on the Applied Biosystems 7500 Real Time PCR System (Applied Biosystems, Foster City, CA) for 40 cycles of 15 s at 95°C, 30 s at 60°C and 30 s at 72°C. Resulting values of mean threshold cycles (Ct) were normalized as previously described^57^. Primers used in real-time PCR analysis were listed in Table S3.

### Vegetative growth, conidiation, and appressorium formation

Vegetative growth rate was measured as colony diameter on complete media, minimal media, oatmeal agar and V8 juice agar plates with at least three replicates. Ability to produce conidia was measured by counting the number of conidia from 8-days-old oatmeal agar in 6-well plates. Conidia were collected by flooding the plate with 5 ml of sterilized distilled water and number of conidia was counted using hemacytometer under a microscope. Conidial germination and appressorium formation were measured on plastic coverslips. Conidia were harvested from 8 to 10-day-old culture on oatmeal agar plate with sterilized distilled water. Conidial suspension of 40 μl was pipetted onto plastic coverslips following adjustment of its concentration to ~2 × 10^4^ spores/ml. Drops were placed in a moistened box and incubated at 25°C. The percentage of conidia germinating and germinated conidia forming appressoria was determined by microscopic examination of at least 100 conidia per replicate in at least two independent experiments with three replicates.

### Pathogenicity assay

Conidia were harvested from 8 to 10-days old culture on oatmeal agar plate with sterilized distilled water. Conidial suspension with concentration adjusted to 5 × 10^4^ spores/ml was spray-inoculated on susceptible rice seedlings (cv. Nagdong) of 3 to 4 leaf stage. Disease lesions were assessed 6 days post inoculation.

### Bisulphite sequencing library construction and high-throughput sequencing

Five microgram of genomic DNA was fragmented by sonication to 100–300 bp with Bioruptor (Diagenode Sparta, NJ). The fragmented DNA was end-repaired and ligated to methylated sequencing adapters provided by Illumina according to manufacturer's instructions (Illumina, San Diego, CA). Bisulphite conversion was carried out with ZYMO EZ DNA Methylation-Gold kit (Zymo Research, Irvine, CA) as described in manufacturer's instructions. Following desalting, size selection, PCR amplification, and second size selection step, the library was sequenced using the Illumina Genome Analyzer (GAIIx) according to manufacturer's instructions. Raw GA sequencing data were processed by Illumina base-calling pipeline.

### Initial processing and mapping of bisulphite sequencing reads

Prior to mapping, short reads from Illumina sequencing were subjected to filtering: i) adapter sequences were trimmed out of reads; ii) reads with N bases over 10% of reads were filtered out; iii) if there were reads with over 10% of bases of read whose qualities are less than 20, these bases were trimmed off from reads.

Due to the nature of bisulphite sequencing, both reads and references genome should be transformed for alignment and mapping as previously described^58^. Observed cytosines on forward read were *in-silico* replaced by thymines and observed guanines on reverse read were *in silico* replaced by adenosines. *M. oryzae* reference genome sequences were downloaded from Magnaporthe comparative database (*Magnaporthe* comparative Sequencing Project, Broad Institute of Harvard and MIT (http://www.broadinstitute.org/)) and computationally converted to alignment target sequences: every cytosine in plus strand was converted to thymine and every guanine in minus strand was converted to adenosine. Alignment of reads to references genome was carried out using SOAP aligner^59^, allowing two mismatches. Hits with a single placement and a clear strand assignment were defined as unambiguous alignment (that is, uniquely mapped reads) and used to compute the coverage and methylation levels of the local region.

#### Bisulphite-PCR validation for target regions

At least two microgram of genomic DNA was fragmented by sonication to 100 bp–500 b with Bioruptor (Diagenode Sparta, NJ) and bisulphite-converted manually as previously described^33^. Primers were designed to amplify target regions of the bisulphite-converted DNA for validation of high-throughput bisulphite-sequencing results. Amplified products were cloned into pGEM-T Easy vector (Promega, Madison, WI) and at least ten independent clones were Sanger-sequenced for each target region. Primers used in validation experiment are listed in Table S3.

### RNA-seq experiment and analysis

RNA-seq was carried out with Illumina platform according to manufacturer's instructions for total RNAs extracted from mycelia tissues (two biological replicates) of wild-type and DMTase mutants. Resulting paired-end sequencing reads were aligned and quantified using TopHat and Cufflinks^60^ with default parameter values. Sequencing reads were first mapped to annotated transcripts in *M. oryzae* genome using TopHat and fed to Cufflinks to estimate transcript abundance and test for differential expression. Gene expression was calculated as FPKM (fragments per kilobase of transcript per million mapped fragments).

## Author Contributions

J.J., S.-Y.P. and A.H. performed the experiments. J.C., G.-W.L. and J.J. analyzed the data. R.A.D. contributed to analysis of data and writing of the manuscript. J.J. and Y.-H.L. designed the study and wrote the manuscript. All authors reviewed the manuscript.

## Supplementary Material

Supplementary Information Supplementary Information

Supplementary Information Supplementary Dataset 1 - List of genes with methylation in mycelia

Supplementary Information Supplementary Dataset 2 - List of genes with methylation in conidia

Supplementary Information Supplementary Dataset 3 - List of genes with methylation in appressoria

Supplementary Information Supplementary Dataset 4 - List of genes with methylation in dim-2
