# Supplementary material for: Genome-wide profiling of DNA methylation provides insights into epigenetic regulation of fungal development in a plant pathogenic fungus, Magnaporthe oryzae
Source: Sci Rep. 2015 Feb 24;5:8567. doi: 10.1038/srep08567 (PMC4338423; doi:10.1038/srep08567)
Supplement: Supplementary Information [file srep08567-s1.pdf]

Supplementary Information

Genome-wide profiling of DNA methylation provides insights into epigenetic regulation of fungal development in a plant pathogenic fungus, *Magnaporthe oryzae*

Junhyun Jeon, Jaeyoung Choi, Gir-Won Lee, Sook-Young Park, Aram Huh, Ralph A. Dean, Yong-Hwan Lee

Table S1. Data statistics after reads alignment of BS-seq

**Sample**	**Raw reads (M)**	**Raw data (Gb)**	**Mapped reads (M)**	**Mapped data (Gb)**	**Average map rate (%)**	**Whole genome average coverage depth (×)**
Mycelia	18.89	1.70	14.58	1.31	77.18	31.98
Conidia	18.89	1.70	15.41	1.39	81.57	33.80
Appressoria	18.89	1.70	14.12	1.27	74.76	30.98
ΔModim-2 mycelia	18.89	1.70	13.72	1.24	72.65	30.10
ΔMorid mycelia	18.89	1.70	11.85	1.07	62.75	26.00

Table S2. List of loci that are validated for DNA methylation in conidia-derived mycelia

Locus		Feature	B[b]	M[c]	B∩M[d]
Chr.[a]	position				
1	4653636-4653982	upstream of MGG_10510	9	9	8
2	5278750-5279211	upstream of MGG_15334	15	18	14
1	821210-821420	Intergenic region	15	16	13
3	5011776-5011934	ORF (MGG16885)	13	15	11
5	1232184-1232244	ORF (MGG00889)[e]	0	0	0
7	Multiple positions including Supercontig 7: 3282757-3282812	a retrotransposon, MAGGY[e]	10	14	9

[a]Chromosome

[b]Number of mC sites in BS-PCR experiment

[c]Number of mC sites in methylC-seq

[d]Number of mC sites that overlap between BS-PCR and methylC-seq

[e]Sequences that are predicted to have no methylation or methylation by Southern blot analysis and used as negative and positive control of the experiment

*Note: BS-PCR and Sanger sequencing for loci listed above were also carried out for conidia, appressoria, and *ΔModim-2*, confirming little or absence of mC sites predicted in methylC-seq.

Table S3. List of primers used in this study

Name	Sequences (5′ →3′)	Use
MGG00889 UF	GTGCAGGTTCGTTCCTACTT	KO[a]
MGG00889 UR	GCACAGGTACACTTGTTTAGAGATCTGATGGTCAAGGTGAGAA	KO
MGG00889 DF	CCTTCAATATCATCTTCTGTCGACATCATTGTCCTGGAAAACA	KO
MGG00889 DR	GGTTGGCTGGCTAAACTAGA	KO
MGG00889 nested F	AATGGATCATCAGCGAAGG	KO
MGG00889 nested R	TGATGGGTTATTCGTAATGGC	KO
MGG00889 RT F	AAGGTAAATCCGACTGAACA	RT
MGG00889 RT R	AGGCTTGCGAGAAATGAC	RT
MGG02795 UF	GCAGGACCGCATCTCGCTCA	KO
MGG02795 UR	GCACAGGTACACTTGTTTAGAGACCCTCGCGGCATCCCTCAG	KO
>MGG02795 DF	CCTTCAATATCATCTTCTGTCGAGTACCATGCGCCTTCCACA	KO
MGG02795 DR	AAGCAGGCTCCGAAGAAGAA	KO
MGG02795 nested F	CATCGAGCGGCGCAAGAAG	KO
MGG02795 nested R	GCCTTTTCGCCACCATTCT	KO
MoDIM-2 RT F	AAGGTAAATCCGACTGAACA	RT[b]
MoDIM-2 RT R	AGGCTTGCGAGAAATGAC	RT
MoRID RT F	CAGCTCTATTGTCATTTGTGGC	RT
MoRID RT R	TCCGAGTCTGCAAAGTTGTC	RT
MGG_889 BS F	TTAAAAAAGGTAAATTTGATTGAAT	BS-PCR[c]
MGG_889 BS R	CAAACCACCTTATATTTCCATA	BS-PCR
MAGGY BS F	GAATATAATTAATGATTTGATTTGA	BS-PCR
MAGGY BS R	TCATACTACTTTAACCAAAACATT	BS-PCR
MGG_10510 P_F	AAAAAATAGGATTATATAAGGAA	BS-PCR
MGG_10510 P_R	TTAAAATATCAAATAATTTATTTTT	BS-PCR
MGG_15334 P_F	AAGGTTGAGTAATTATTGGTAA	BS-PCR
MGG_15334 P_R	CCACAACTTTATAATAATTTTTC	BS-PCR
MGG_16073 ORF F	ATGTTAATAATAATGGTGTTAATAG	BS-PCR
MGG_16073 ORF R	CTACCCTATACCAAATTTAATAC	BS-PCR
MGG_16885 ORF F	TATTAAATTTGGTATAGGGTAGT	BS-PCR
MGG_16885 ORF R	TTATTTTACATATAATAAACCCAC	BS-PCR
Chr1_Ig F	GTTATTATGTTTGATAGTGATTATT	BS-PCR
Chr1_Ig R	TACATACTCACAATTTACAAAAA	BS-PCR

[a]Gene knockout

[b]Real-time PCR

[c]Sanger sequencing of PCR product using bisulphite-treated genomic DNA as template

Figure S1

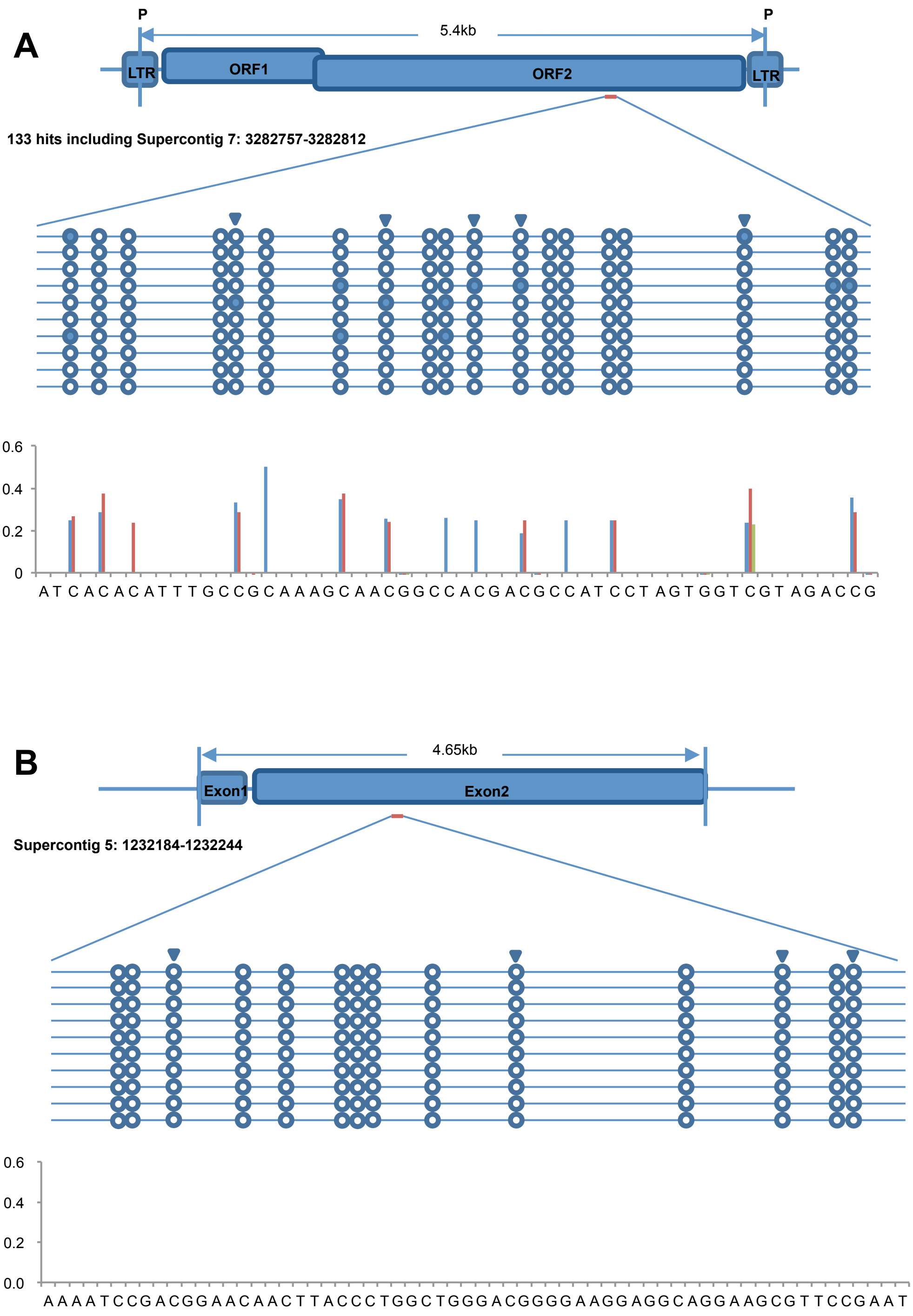

Figure S1. Representative regions in which DNA methylation predicted in bisulfite sequencing (BS -seq) were validated in our study. DNA methylation from Sanger sequencing of bisulfite-PCR (line s and circles) and BS-seq (bar graph) in one MAGGY locus (**A**) and MGG00889 (**B**). Each line rep resents a Sanger sequencing result for loci indicated by the red bar below the ORF diagram. Empty and filled circles indicate non-methylated and methylated cytosines, respectively. Triangles indicate the positions of cytosines in a CG context.

Figure S2

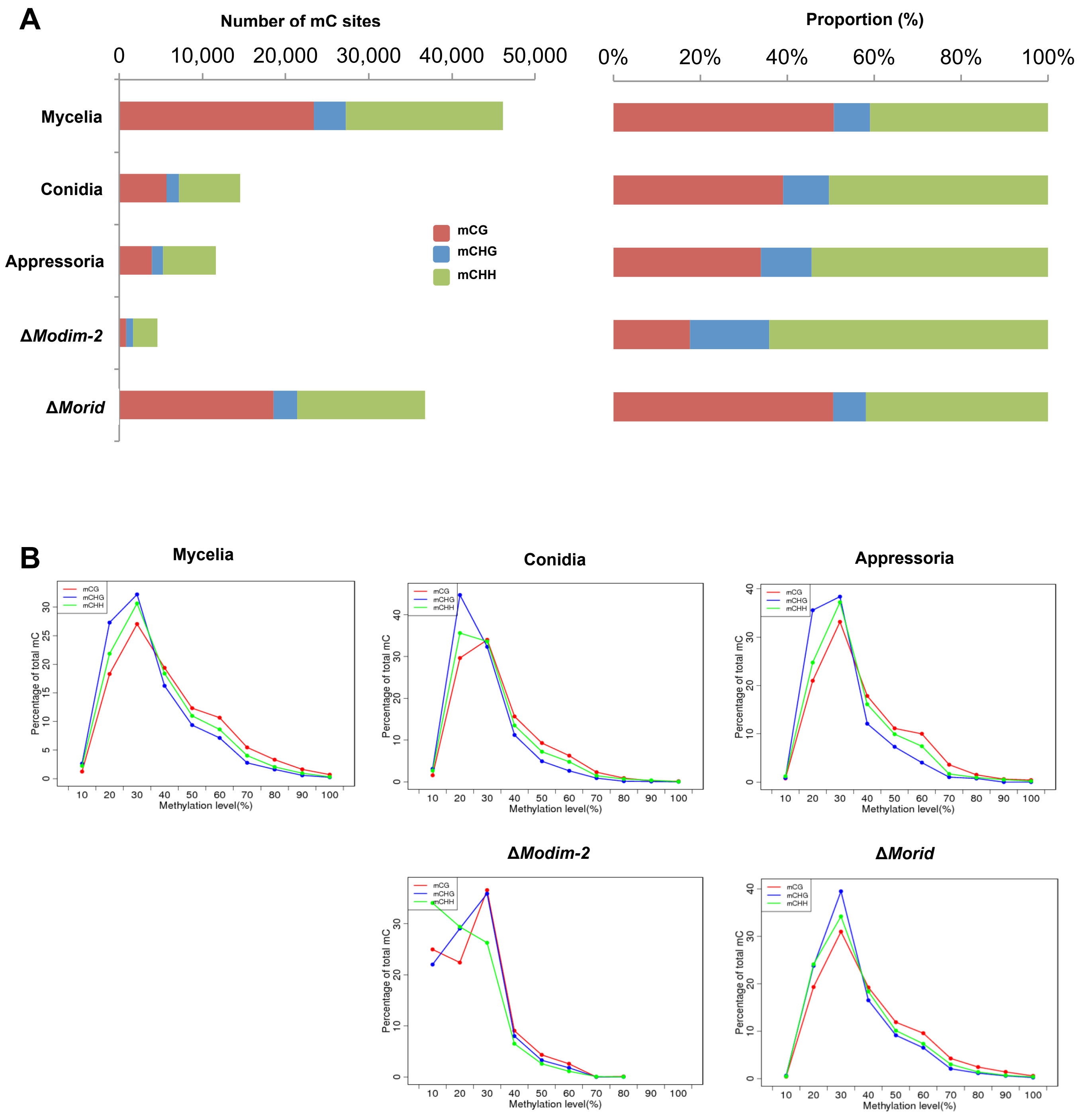

Figure S2. Summary of DNA methylation in the genome of *Magnaporthe oryzae*. (**A**) Number (left) and proportion (right) of methylcytosine (mC) sites in different sequence contexts identified in each sample subjected to bisulfite sequencing (BS-seq). (**B**) Distribution of the average methylation level of individual mC sites. The *x*-axis represents the methylation level of mC divided into 10 intervals. The *y*-axis represents the percentage of mCs that fall into methylation-level bins.

Figure S3

Density of mCGs on the chromosomes (mycelia)

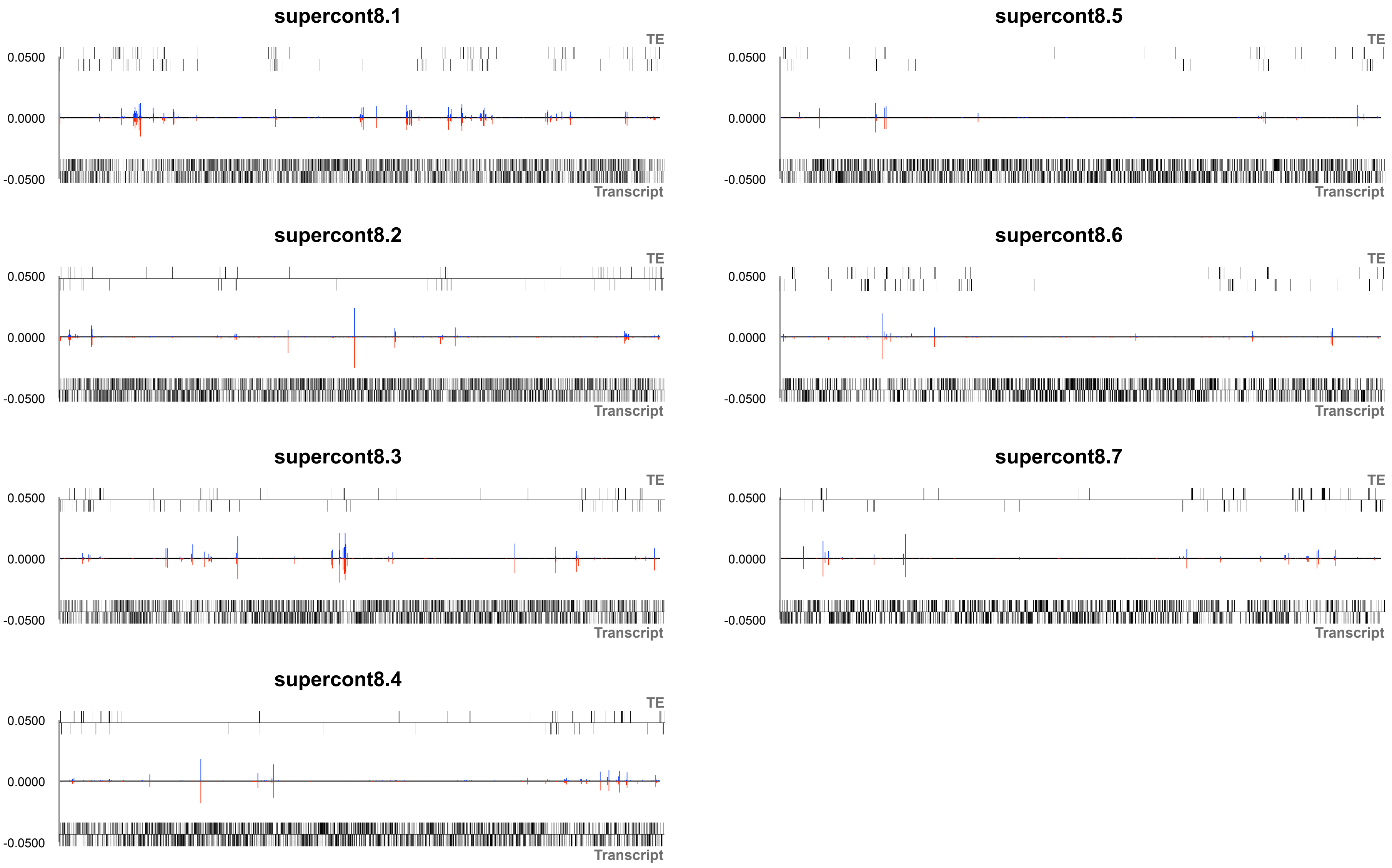

Figure S3

Density of mCHGs on the chromosomes (mycelia)

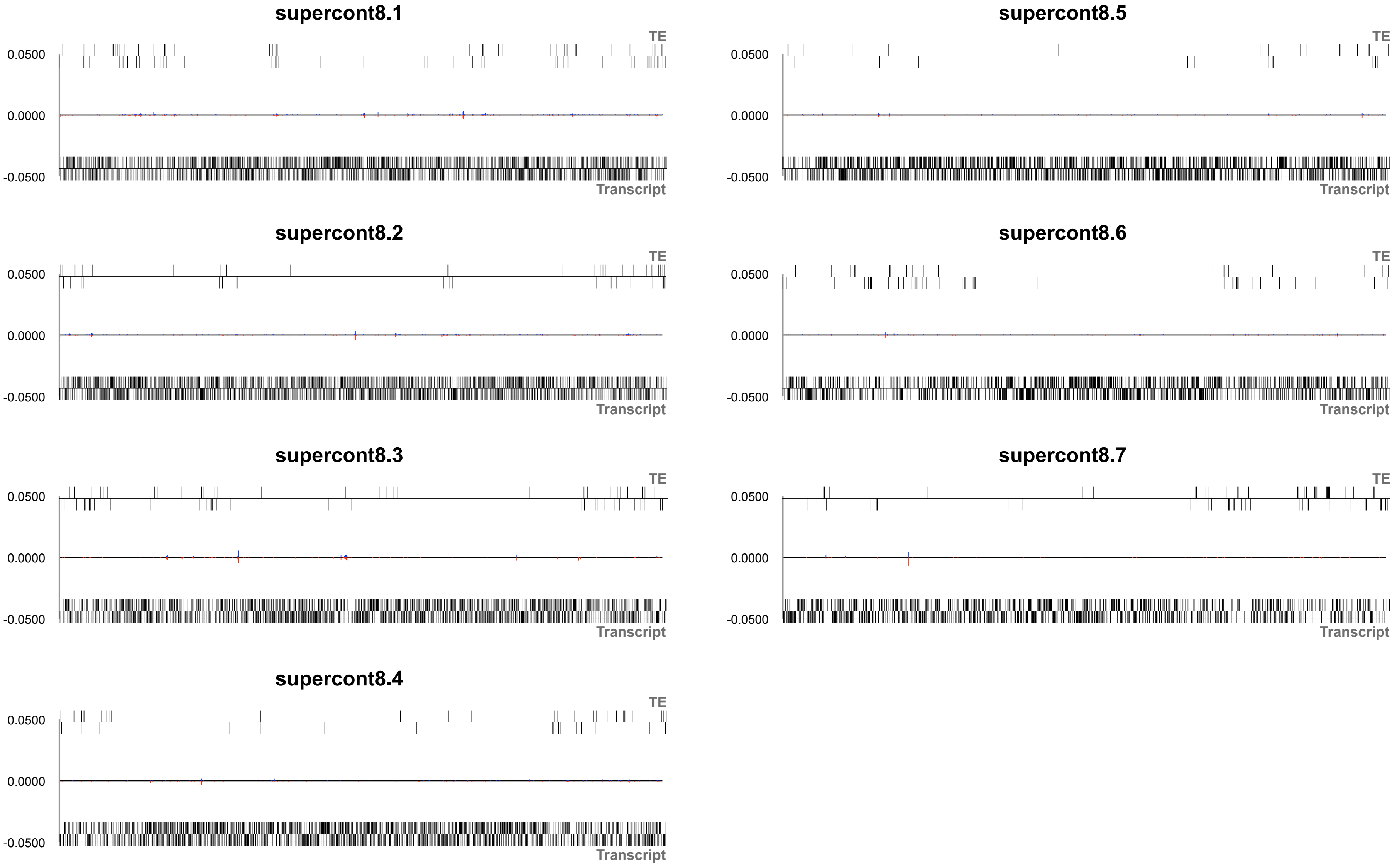

Figure S3

Density of mCHHs on the chromosomes (mycelia)

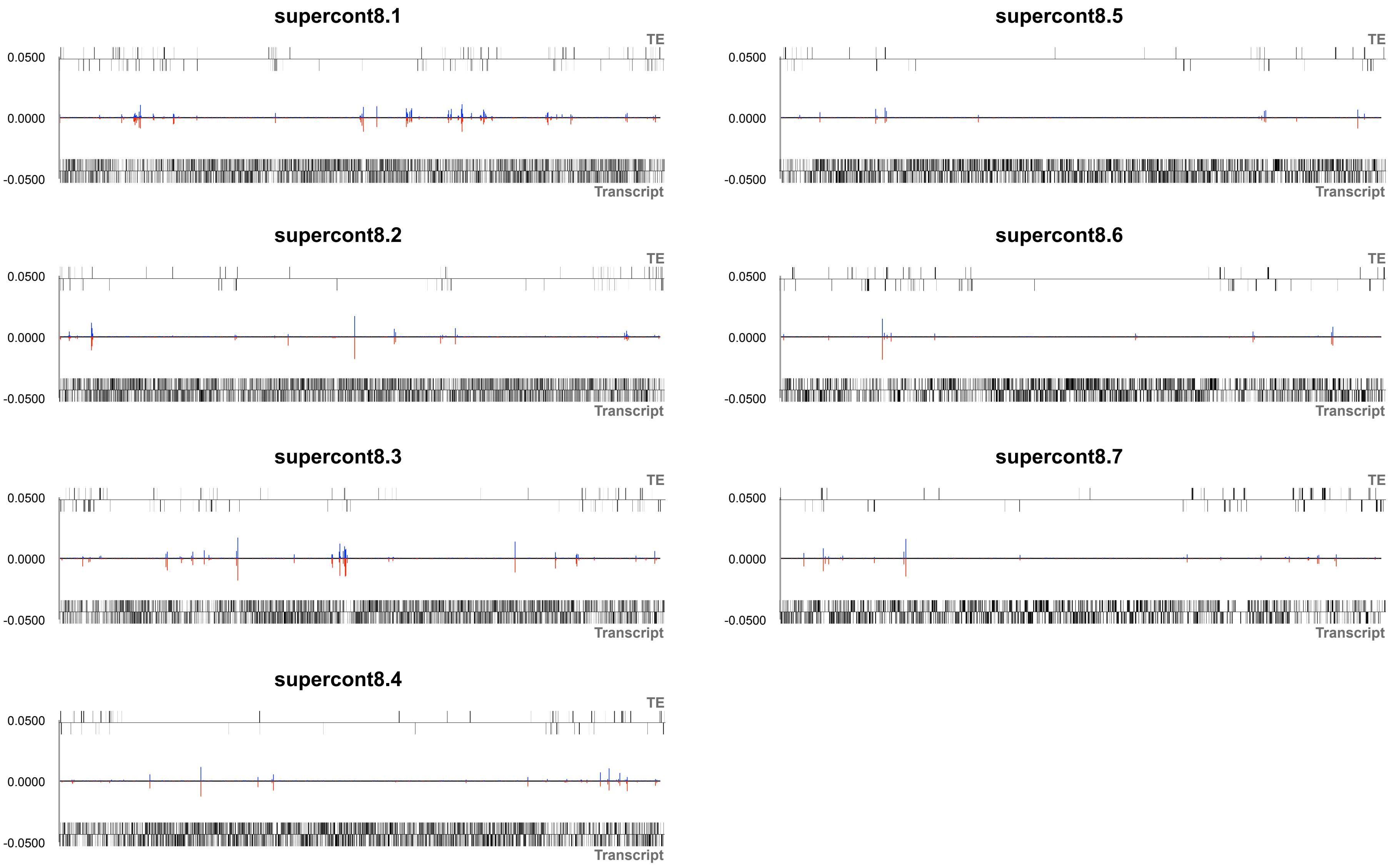

Figure S3

Density of mCGs on the chromosomes (conidia)

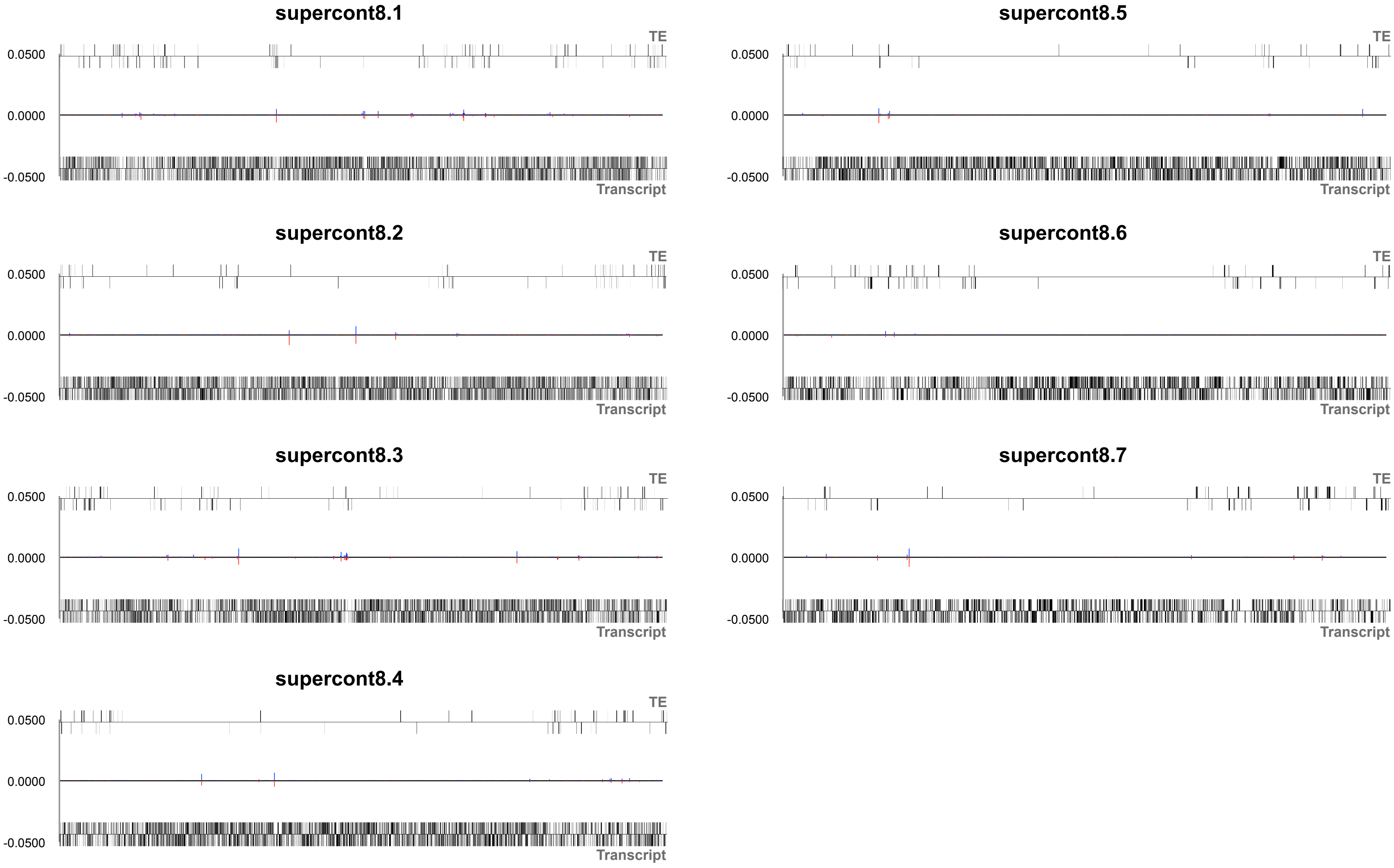

Figure S3

Density of mCHGs on the chromosomes (conidia)

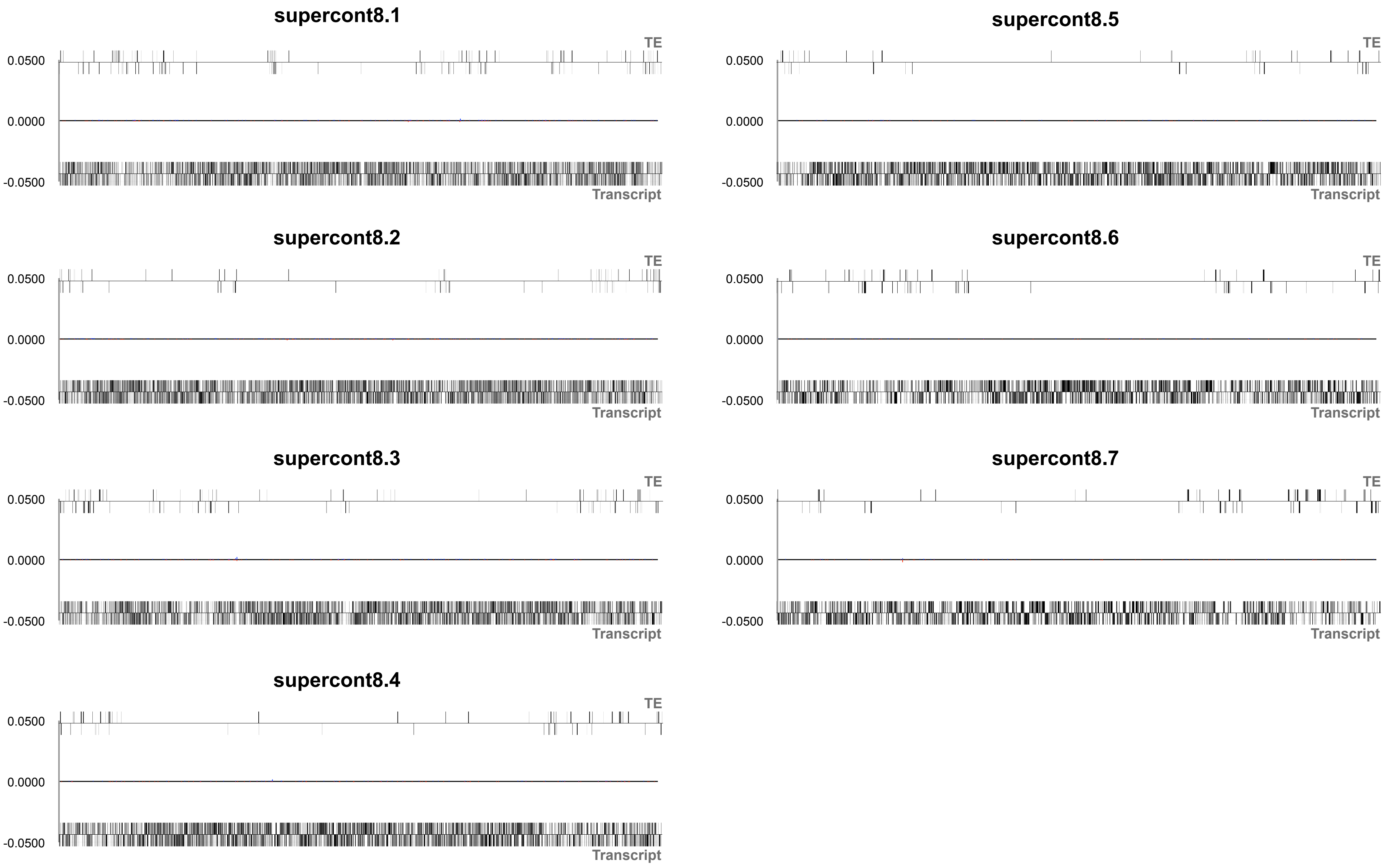

Figure S3

Density of mCHHs on the chromosomes (conidia)

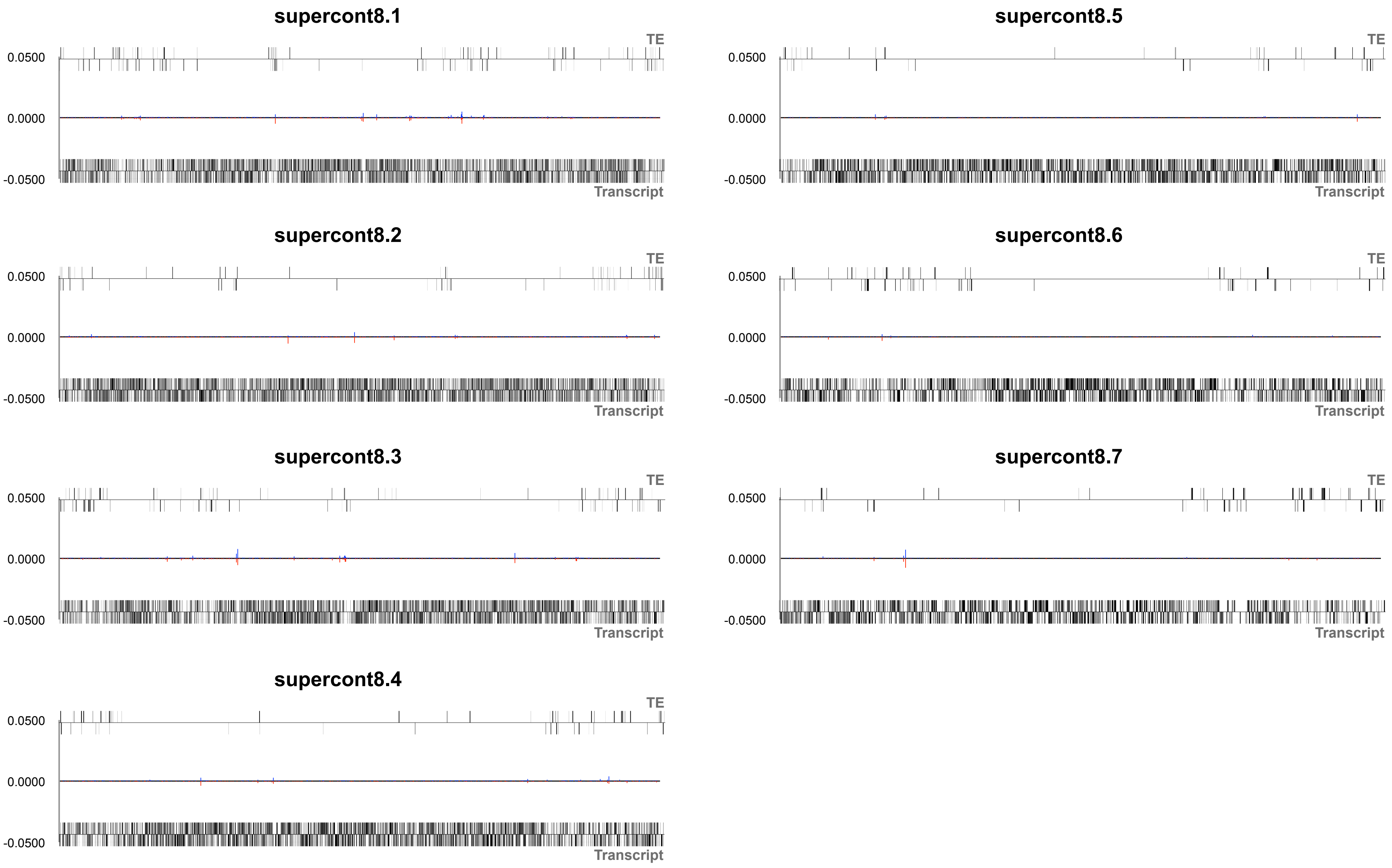

Figure S3

Density of mCGs on the chromosomes (appressorium)

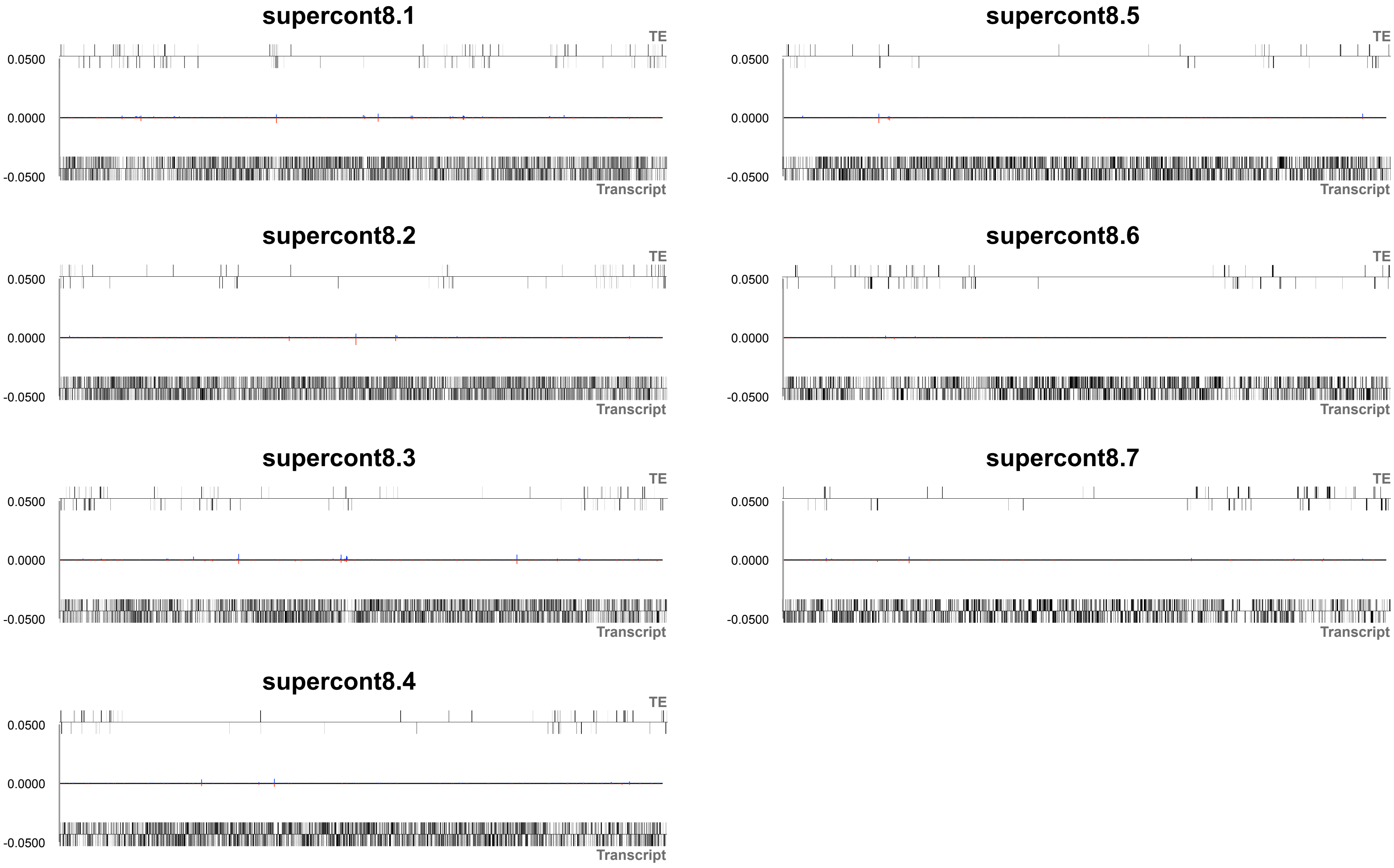

Figure S3

Density of mCHGs on the chromosomes (appressorium)

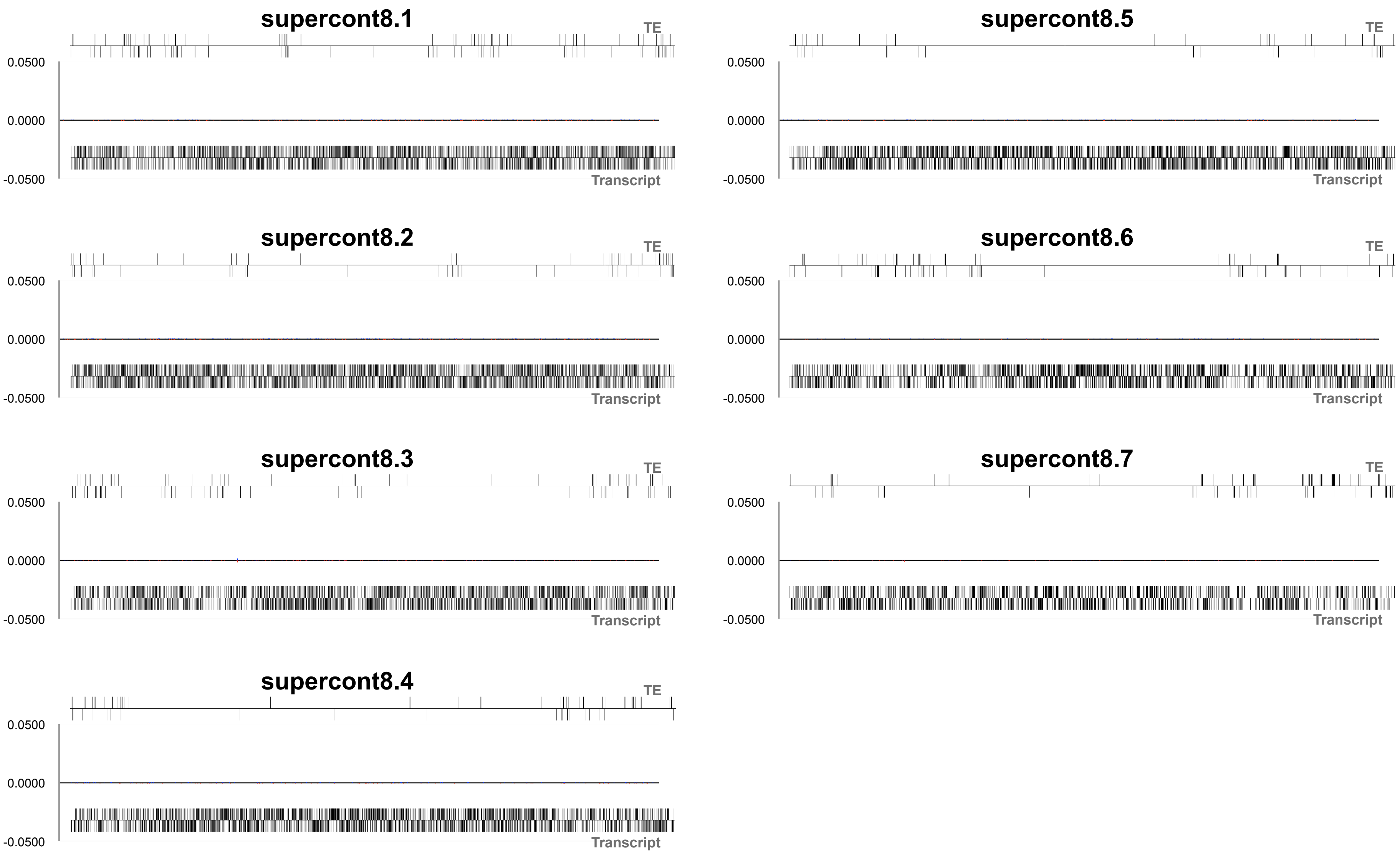

Figure S3

Density of mCHHs on the chromosomes (appressorium)

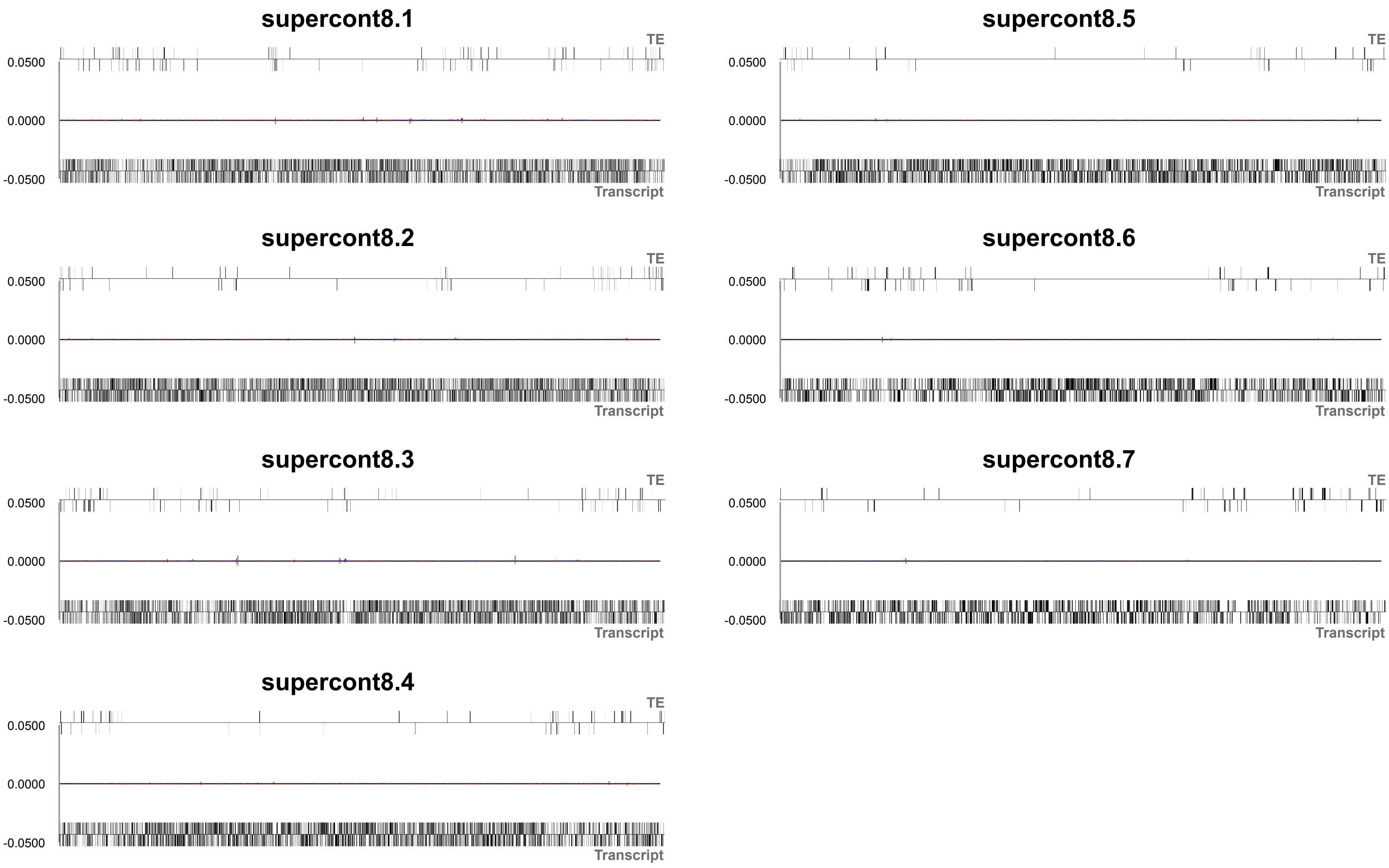

Figure S3

Density of mCGs on the chromosomes (dim-2)

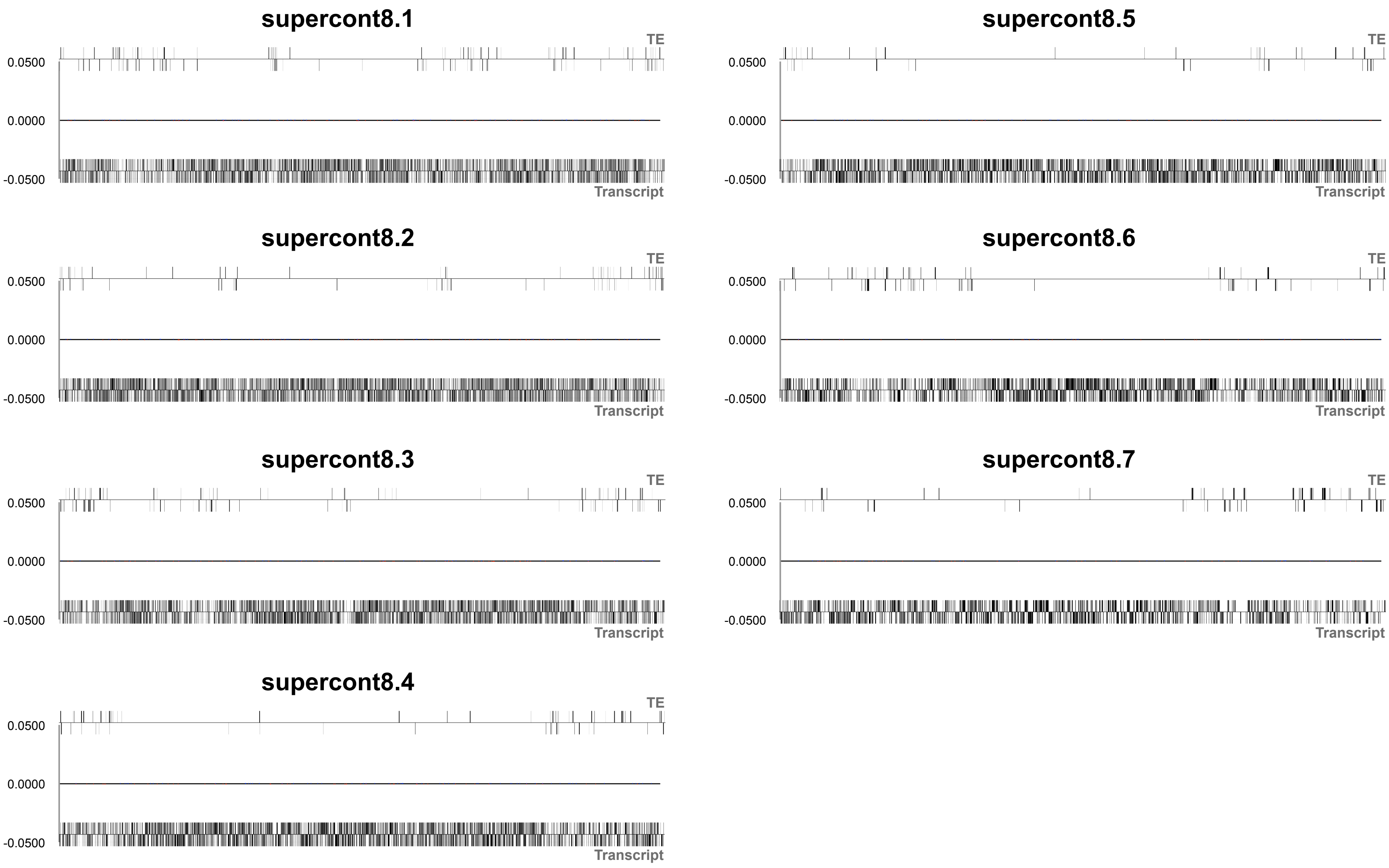

Figure S3

Density of mCHGs on the chromosomes (dim-2)

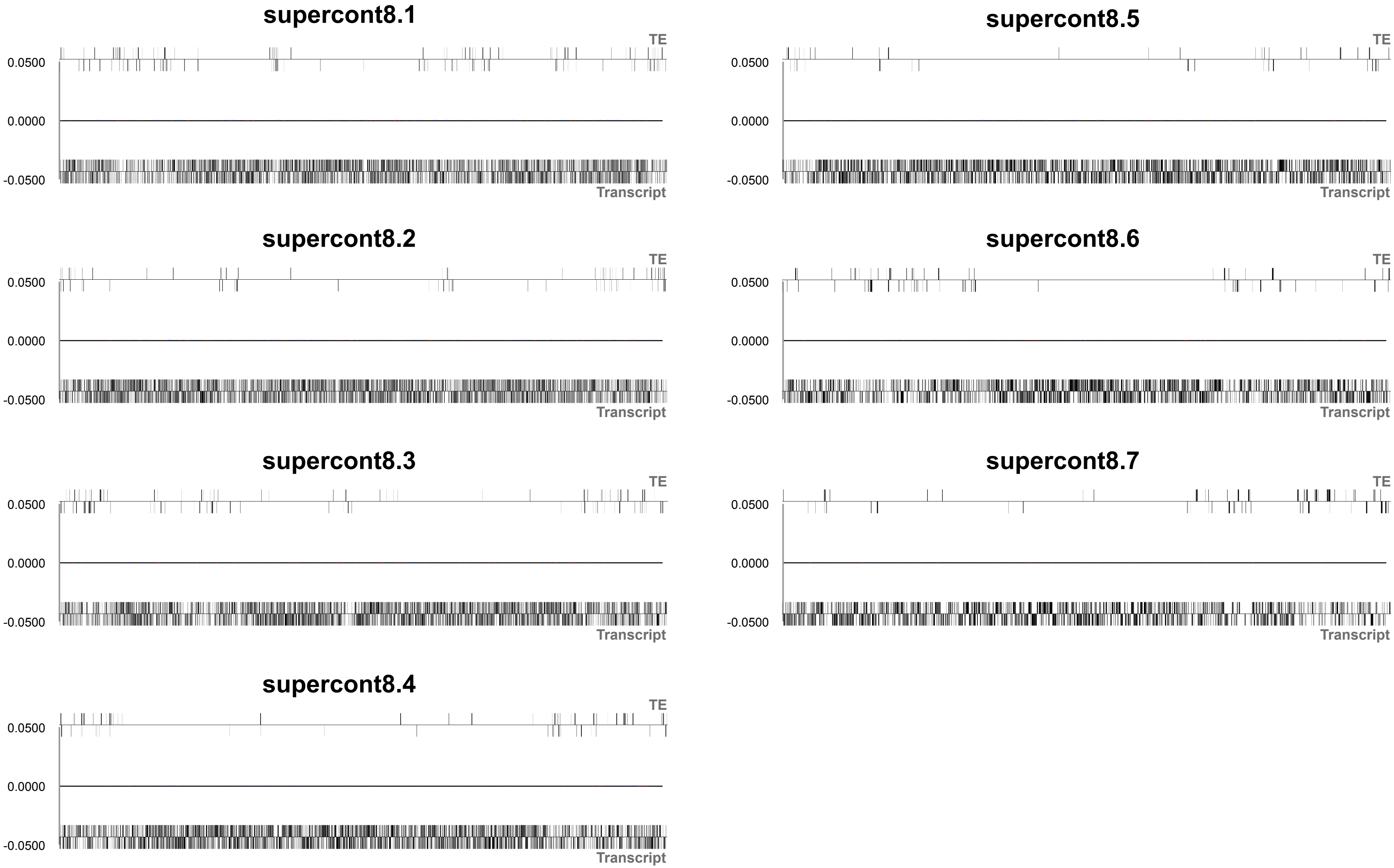

Figure S3

Density of mCHHs on the chromosomes (dim-2)

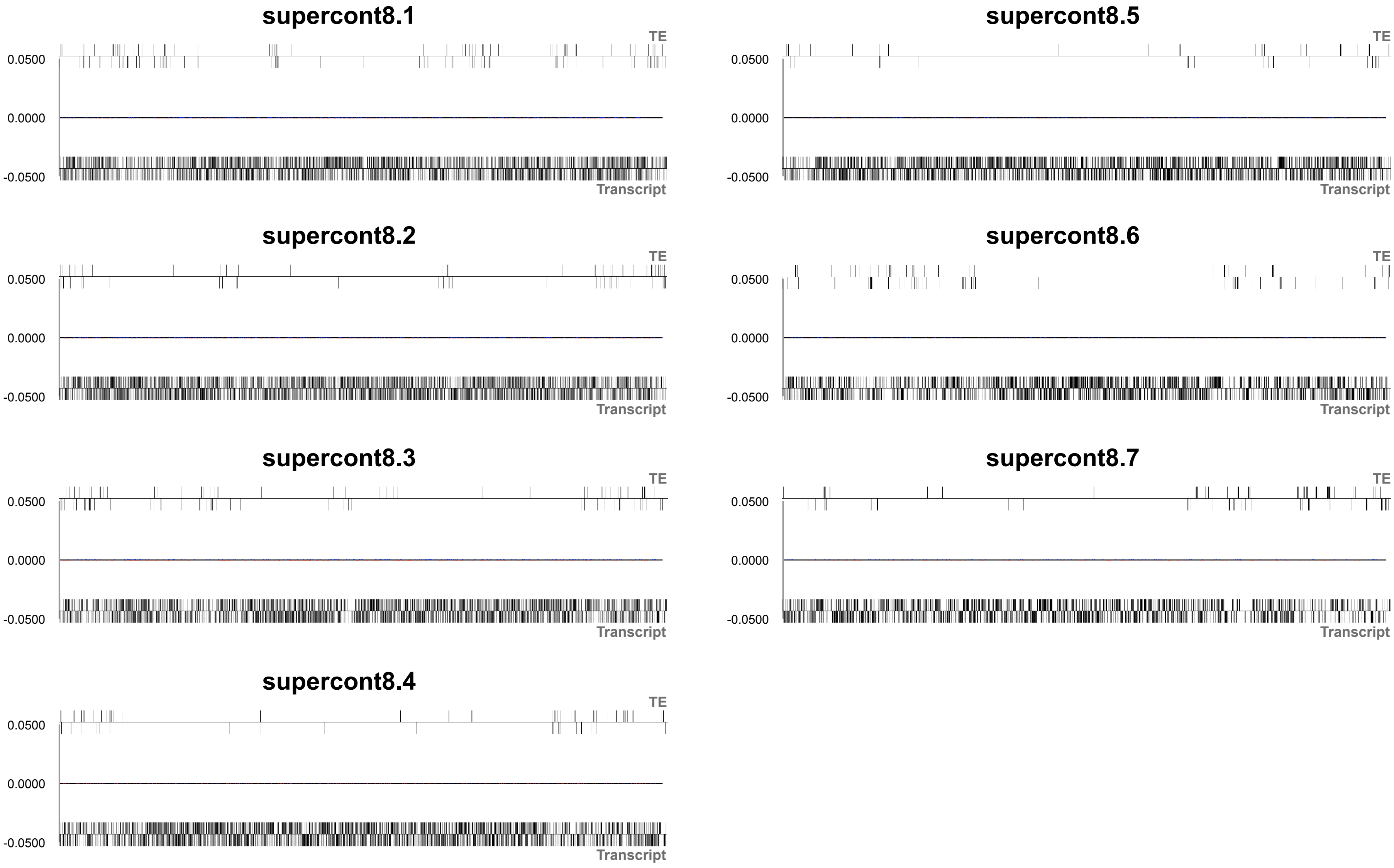

Figure S3

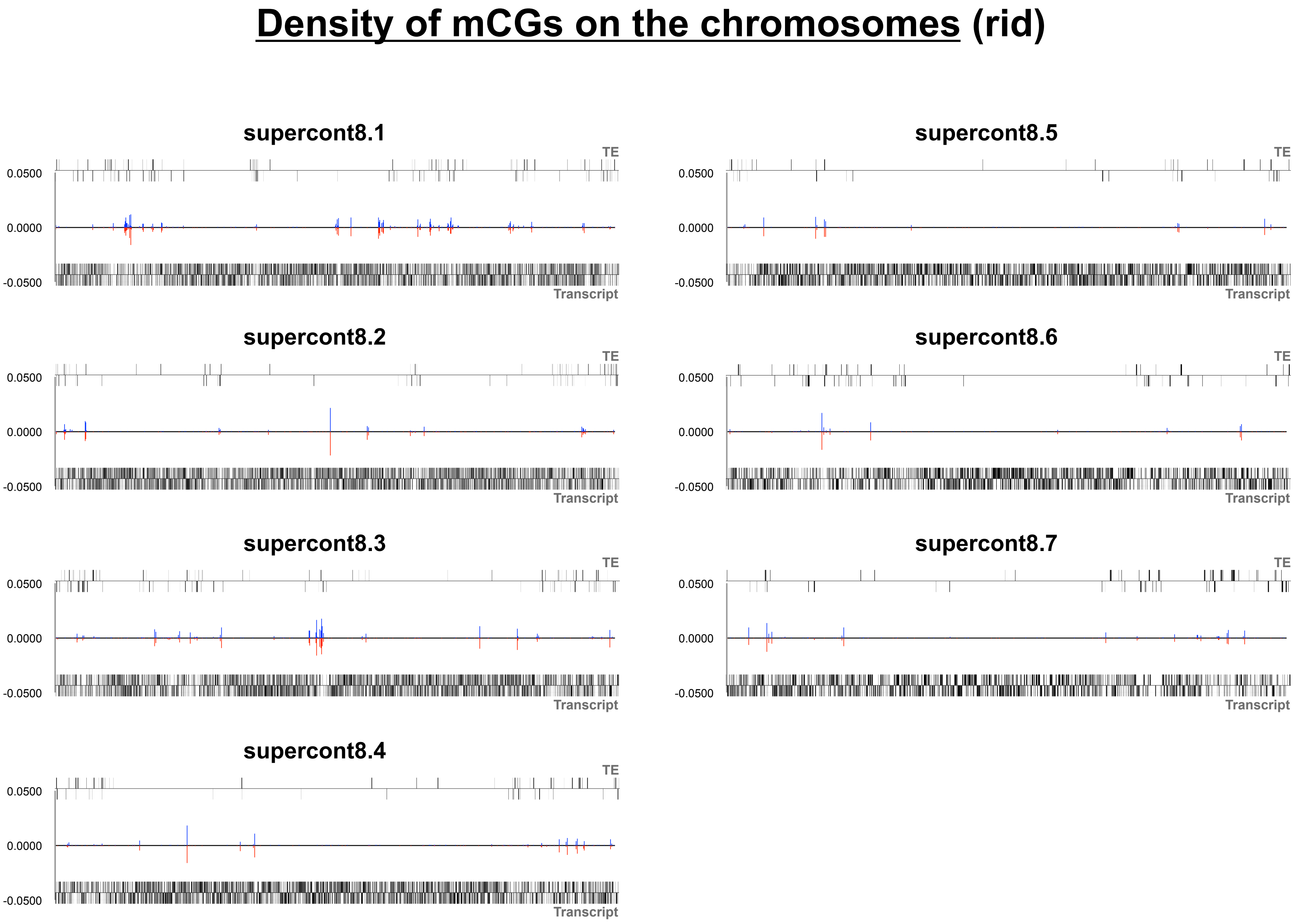
Density of mCGs on the chromosomes (rid)
supercont8.1
supercont8.2
supercont8.3
supercont8.4
supercont8.5
supercont8.6
supercont8.7
TE
Transcript
0.0500
0.0000
-0.0500

Figure S3

Density of mCHGs on the chromosomes (rid)

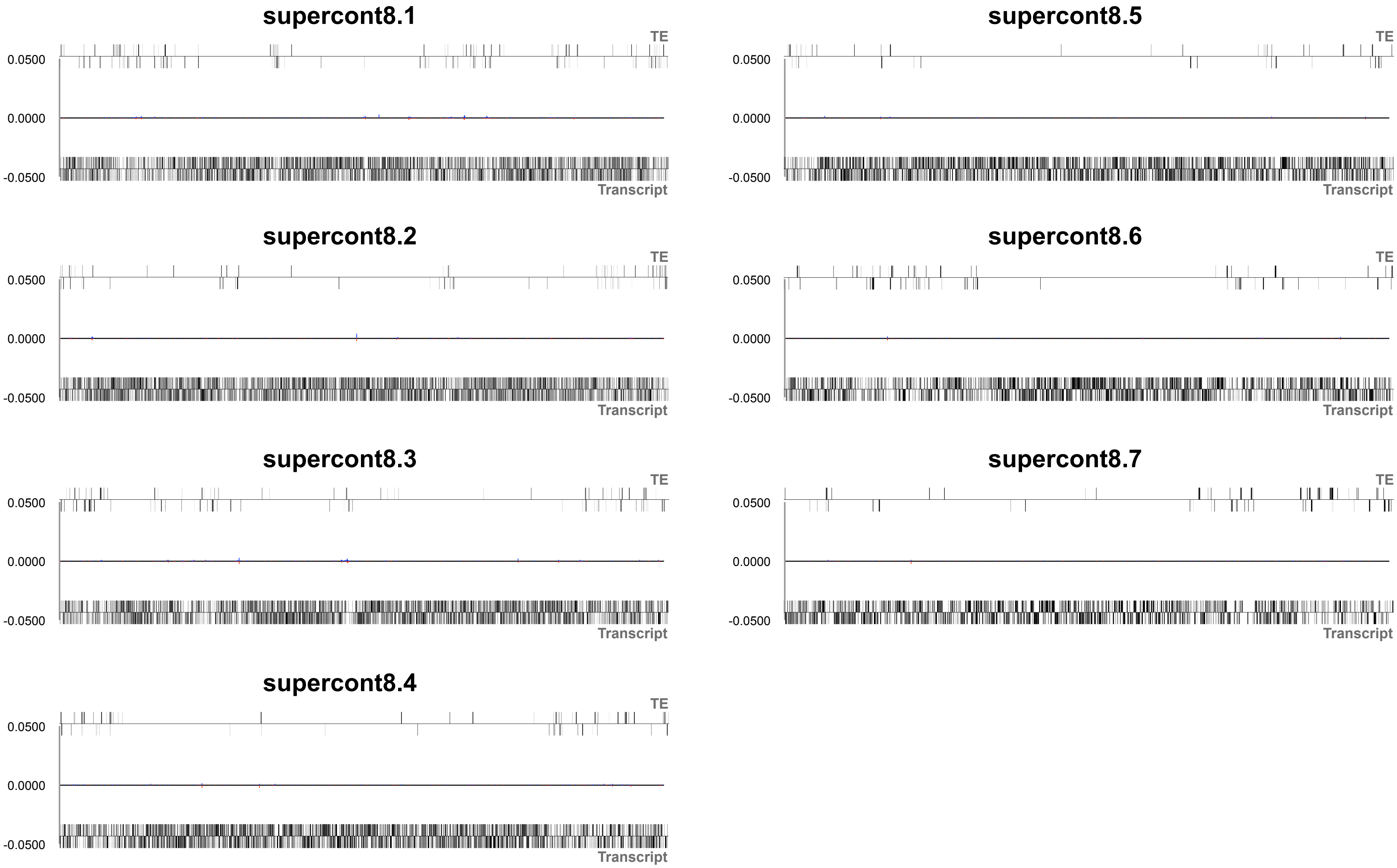

Figure S3

Density of mCHHs on the chromosomes (rid)

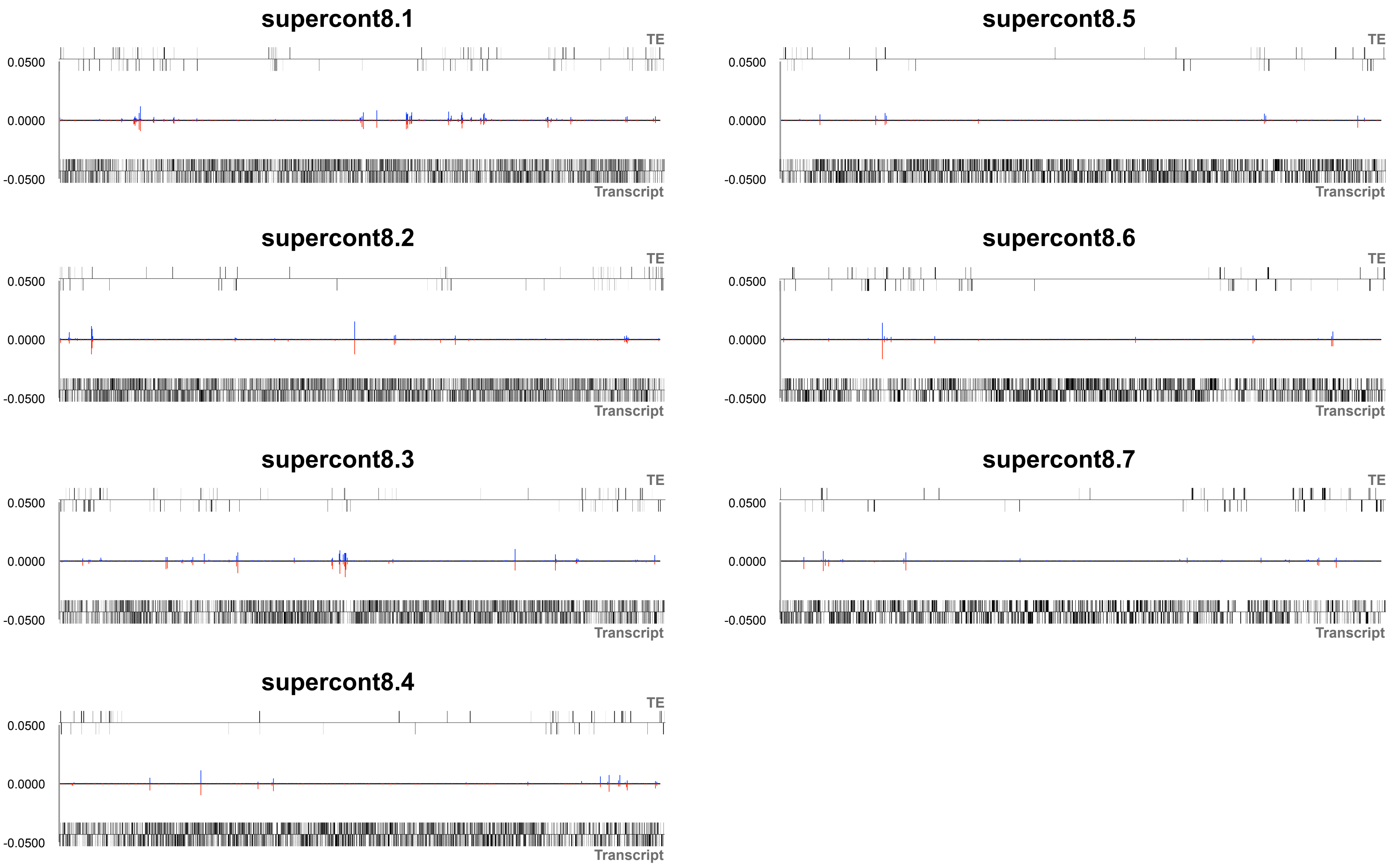

Figure S3. Chromosomal distribution of DNA methylation in the genome of *M. oryzae*. The density of methylcytosines (mCs) identified on each strand throughout chromosomes of each sample depending on sequence contexts was calculated and plotted in 10kb bin (middle bar). Blue and red tick marks indicate methylation density in Watson and Crick strand, respectively. Dark grey tick marks at the top and bottom of plot indicate density of genes and transposable elements, respectively.

Figure S4

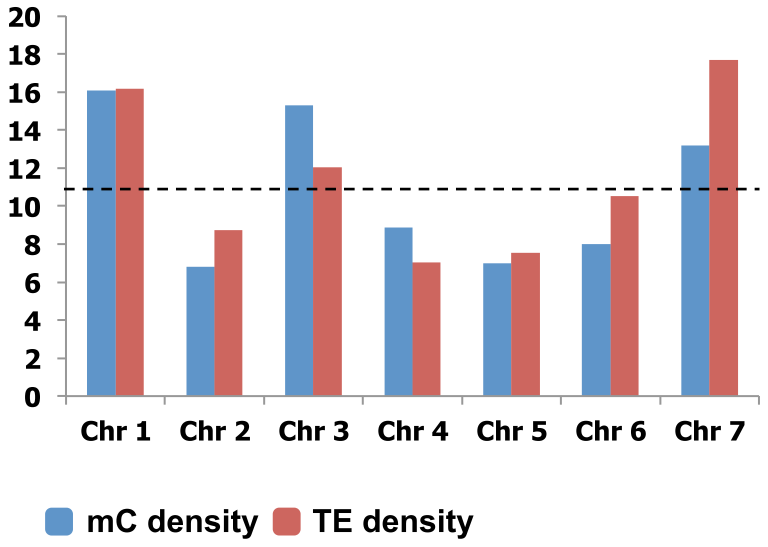

Figure S4. Density of methylcytosine (mC) sites and transposable elements (TEs) over chromosomes. The blue bar represents the number of mC sites per 10 kb in each chromosome, and the red bar represents the density of TEs in each chromosome ((total length of TEs/length of the corresponding chromosome) × 1000). The dotted line indicates the expected mC density across chromosomes. Unassigned genome sequences are not included.

Figure S5

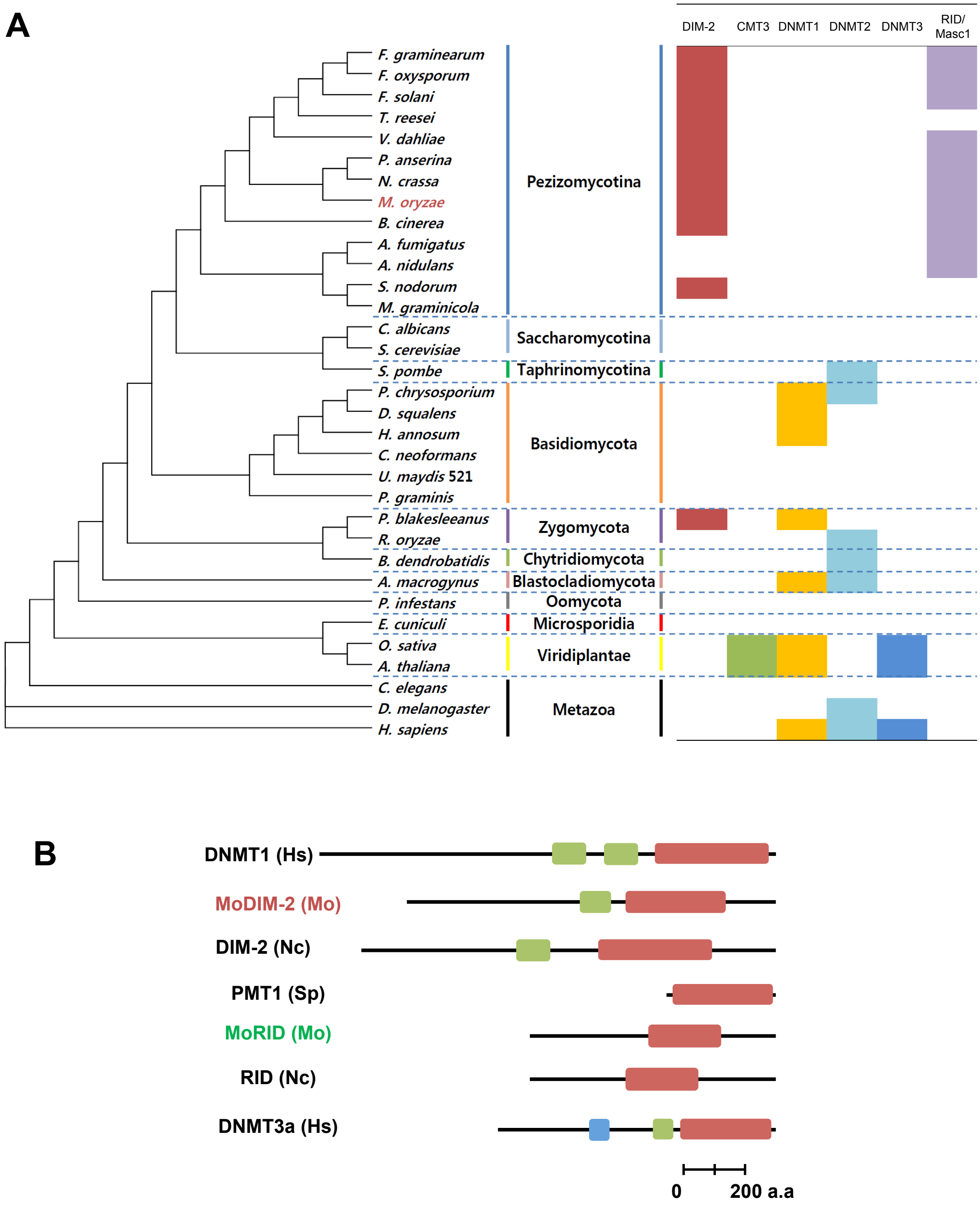

Figure S5. DNA methyltransferases in diverse organisms. (**A**) Kingdom-wide distribution of DNA methyltransferases. The cladogram was constructed for representative species of each phylum by CVtree with the following options: K-tuple length = 7 and sequence type = amino acid (http://tlife.fudan.edu.cn/cvtree/). The presence (colored box) or absence (box with no color) of each family of DNA methyltransferases in different taxa was determined using the BLAST matrix function embedded in CFGP (http://cfgp.riceblast.snu.ac.kr/main.php). To remove spurious hits, an e-value cutoff of 1e–10^{-5} and a score cutoff of 100 were initially used; the remaining hits were examined manually. (**B**) Domain architecture of representative DNA methyltransferases. Light green, BAH domain; blue, PWWP domain; red, DNA methyltransferase domain.

Figure S6

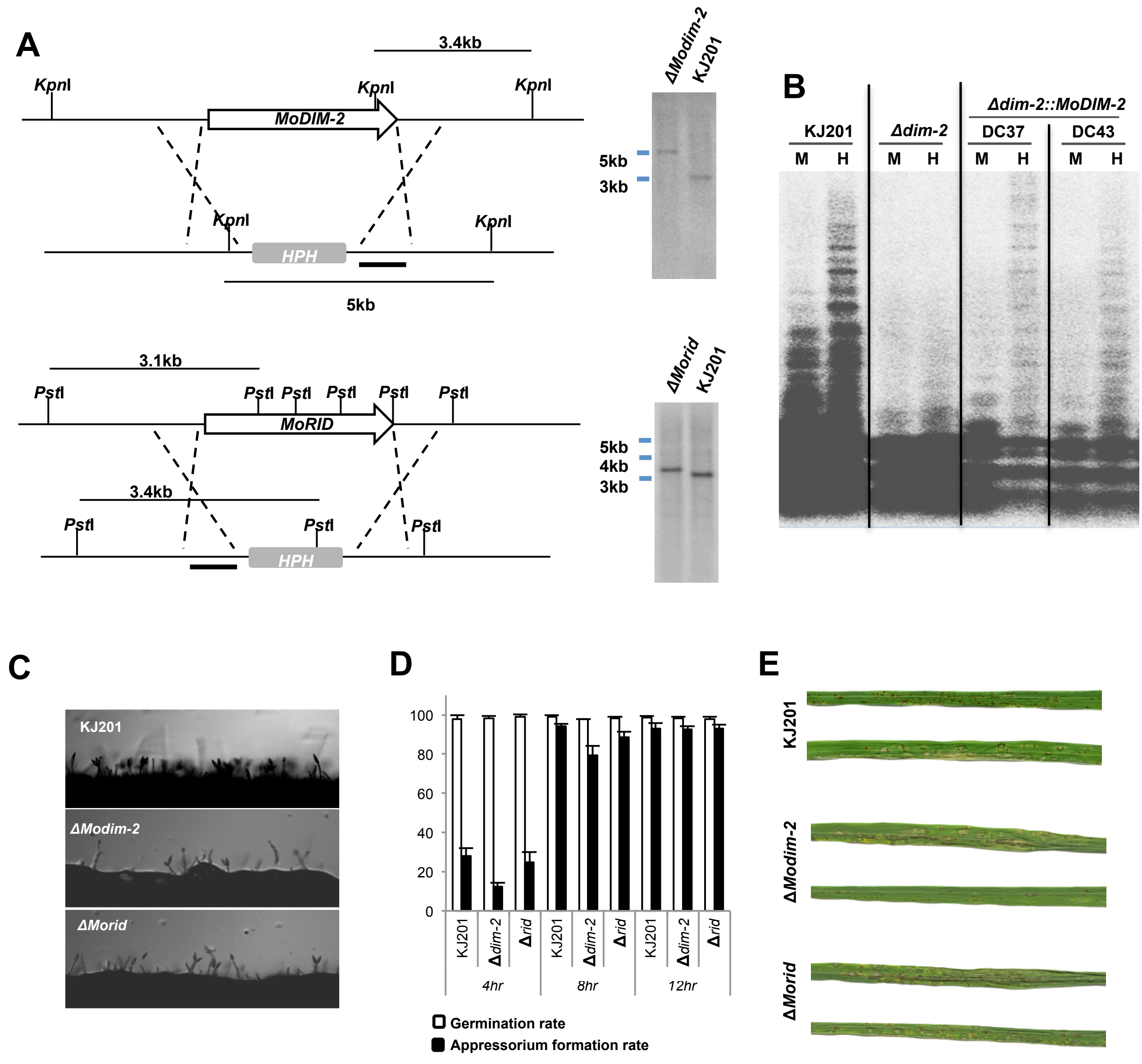

Figure S6. Deletion of genes encoding DNA methyltransferases in *Magnaporthe oryzae*. (**A**) So uthern blot analysis of gene deletion mutants for *MoDIM-2* (top) and *MoRID* (bottom). A schema tic diagram depicting gene deletion via double homologous recombination is shown on the left a nd the result of Southern hybridization using sequences indicated as a black bar as a probe is sho wn on the right. (**B**) Complementation of methylation defects in Δ*Modim-2* by the introduction o f a native *MoDIM-2* copy (H, *Hpa*II; M, *Msp*I). Four separate parts of a single blot (delineated by black vertical lines) were vertically sliced and juxtaposed for qualitative comparison for clarity. (**C**) Conidiophore development in the mutants (8 h post-surface scraping). (**D**) Germination and a ppressorium formation at 4, 8, and 12 h postinoculation (hpi). (E) Pathogenicity towards rice pla nts (6 days postinoculation).

Figure S7

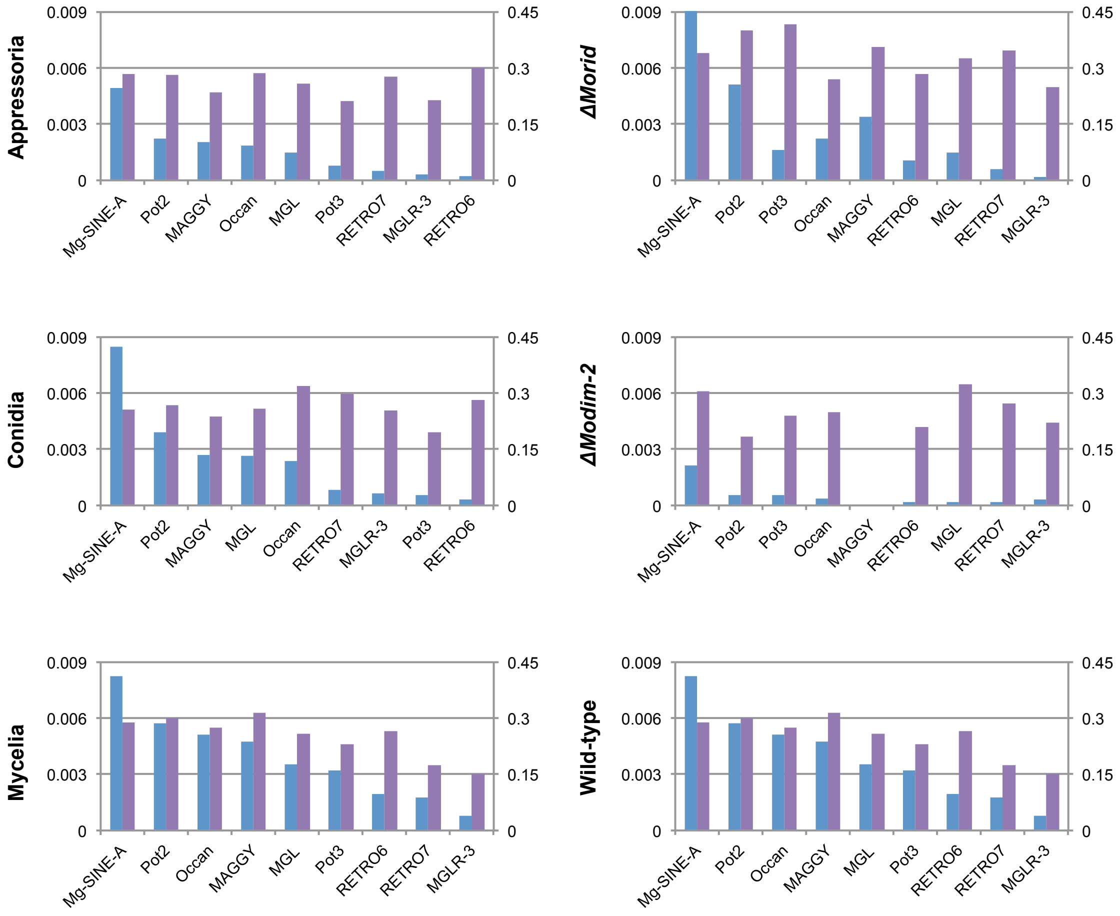

Figure S7. Density (blue bar) and average methylation level (purple bar) of methylcytosine (mC) sites in different transposable elements (TEs). TEs are arranged on the *x*-axis in ascending order of length.
